# Cocrystals of a coumarin derivative: an efficient approach towards anti-leishmanial cocrystals against MIL-resistant *Leishmania tropica*

**DOI:** 10.1107/S2052252524001416

**Published:** 2024-03-01

**Authors:** Muhammad Shahbaz, Saba Farooq, M. Iqbal Choudhary, Sammer Yousuf

**Affiliations:** a H.E.J. Research Institute of Chemistry, International Center for Chemical and Biological Sciences, University of Karachi, Karachi 75270, Pakistan; b Dr Panjwani Center for Molecular Medicine and Drug Research, International Center for Chemical and Biological Sciences, University of Karachi, Karachi 75270, Pakistan; Université de Sherbrooke, Canada

**Keywords:** anti-leishmanial, resistant *Leishmania tropica*, cocrystals, crystal engineering, coumarin-3-carb­oxy­lic acid

## Abstract

Leishmaniasis is a neglected parasitic tropical disease with numerous clinical manifestations. One of the causative agents of cutaneous leishmaniasis (CL) is *Leishmania tropica* (*L. tropica*) known for causing ulcerative lesions on the skin. The adverse effects of the recommended available drugs, such as amphotericin B and pentavalent antimonial, and the emergence of drug resistance in parasites, mean the search for new safe and effective anti-leishmanial agents is crucial. Miltefosine (MIL) was the first recommended oral medication, but its use is now limited because of the rapid emergence of resistance. Pharmaceutical cocrystallization is an effective method to improve the physicochemical and biological properties of active pharmaceutical ingredients (APIs). Herein, we describe the cocrystallization of coumarin-3-carb­oxy­lic acid (**CU**, **1a**; 2-oxobenzo­pyrane-3-carb­oxy­lic acid, C_10_H_6_O_4_) with five coformers [2-amino-3-bromo­pyridine (**1b**), 2-amino-5-(tri­fluoro­methyl)-pyridine (**1c**), 2-amino-6-methyl­pyridine (**1d**), *p*-amino­benzoic acid (**1e**) and amitrole (**1f**)] in a 1:1 stoichiometric ratio via the neat grinding method. The cocrystals **2**–**6** obtained were characterized via single-crystal X-ray diffraction, powder X-ray diffraction, differential scanning calorimetry and thermogravimetric analysis, as well as Fourier transform infrared spectroscopy. Non-covalent interactions, such as van der Waals, hydrogen bonding, C—H⋯π and π⋯π interactions contribute significantly towards the packing of a crystal structure and alter the physicochemical and biological activity of **CU**. In this research, newly synthesized cocrystals were evaluated for their anti-leishmanial activity against the MIL-resistant *L. tropica* and cytotoxicity against the 3T3 (normal fibroblast) cell line. Among the non-cytotoxic cocrystals synthesized (**2**–**6**), **CU**:**1b** (**2**, IC_50_ = 61.83 ± 0.59 µ*M*), **CU**:**1c** (**3**, 125.7 ± 1.15 µ*M*) and **CU**:**1d** (**4**, 48.71 ± 0.75 µ*M*) appeared to be potent anti-leishmanial agents and showed several-fold more anti-leishmanial potential than the tested standard drug (MIL, IC_50_ = 169.55 ± 0.078 µ*M*). The results indicate that cocrystals **2**–**4** are promising anti-leishmanial agents which require further exploration.

## Abstract 2

This study demonstrates the synthesis of non-cytotoxic active candidates (co-crystals) of coumarin-3-carb­oxy­lic acid with various coformers to target the MIL-resistant *Leishmania tropica*. These promising anti-leishmanial results indicate the importance of crystal engineering by highlighting that manipulation of supramolecular architecture in the solid state can impact the biological response.

## 1. Introduction

Leishmaniasis is one of the several neglected tropical diseases (NTDs) and the ninth most burdened among infectious diseases (Berry & Berrang-Ford, 2016 ▸). It is caused by the parasitic protozoan leishmanial and transferred to mammals by the bite of a female phlebotomine sandfly vector (Desjeux, 1992 ▸). Every year, approximately 700 000 to 1 000 000 new cases of leishmaniasis are reported (WHO, 2022 ▸). In the last 70 years, no major modifications have been made in the treatment (oral or topical application) or prevention (vaccination) of leishmaniasis (Rezvan & Moafi, 2015 ▸). The available drugs have associated limitations involving toxicity, hydro­phobicity and high cost (Castro-Gomes *et al.*, 2009 ▸; Haldar *et al.*, 2011 ▸). The situation is motivation for many chemists to design structurally diverse libraries to identify promising hits against leishmaniasis (Hussain *et al.*, 2014 ▸). In addition, the increase in the number of outbreaks and scarcity of safe and effective therapies against cutaneous leishmaniasis (CL) demand urgent action to develop new anti-leishmanial agents. Several natural products (Boluda *et al.*, 2007 ▸), sulfonamide analogs (Pinheiro *et al.*, 2019 ▸) and nitro­gen-containing heterocycles (Hussain *et al.*, 2014 ▸; Chen *et al.*, 2008 ▸) are being explored. The toxicity and high cost of existing anti-leishmanial drugs (miltefosine, pentamidine, antimonials and amphotericin B) not only contribute towards economic burden but also prompted an energetic search for more effective treatment from a range of resources available, such as naturally occurring compounds, repurposing of the current drugs and structural modification of drug candidates.

The fundamental physicochemical and biological properties of compounds are associated with the structural features and molecular configuration in the solid state. A change in the functional group, molecular arrangement or interactions exerts a direct effect on the properties of a solid (Seddon & Zaworotko, 1999 ▸). Cocrystals are composed of two or more neutral molecules in a crystal structure with a distinct stoichiometry. They are formed via non-covalent interactions such as hydrogen bonding, π-stacking and van der Waals forces, as well as halogen bonding (Mandal *et al.*, 2019 ▸; Bauzá *et al.*, 2016 ▸). Crystal engineering is a well established approach for designing organic solids with a wide range of applications in the field of pharmaceutical sciences (Moorthy *et al.*, 2010 ▸). Pharmaceutical cocrystals are often designed based on crystal engineering approaches that are effective in improving physicochemical properties of clinical relevance (Desiraju & Parshall, 1989 ▸; Bolla & Nangia, 2016 ▸; Malamatari *et al.*, 2017 ▸). Moreover, the US FDA’s consideration of cocrystals as new and legitimate forms of active pharmaceutical ingredients (APIs) further contributed to a rise in the interest of pharmaceutical manufacturers towards the development of certain cocrystals as new drug leads (Kale *et al.*, 2017 ▸). In the literature there are many studies that report improvement in the biological activity of pharmaceutical ingredients via cocrystallization (Aakeröy *et al.*, 2011 ▸; Nascimento *et al.*, 2021 ▸). Pharmaceutical cocrystals have been widely used in industries and academia in the last two decades to improve the ADME (absorption, distribution, metabolism and excretion) properties of APIs, as well as bioavailability, solubility, chemical stability, hygrostability, dissolution rate, tabletability *etc.* (Thakuria *et al.*, 2013 ▸; Kumari & Ghosh, 2020 ▸; Box *et al.*, 2016 ▸). As a result, numerous studies covering the fundamental aspects of cocrystallization have been published. The literature provides several examples of cocrystals, such as ertuglifozin L-pyroglutamic acid, sacubitril-valsartan, escitalopram oxalate-oxalic acid and termidol-celecoxib that are currently on the market or in various clinical trial phases (Kaduk *et al.*, 2021 ▸; Videla *et al.*, 2017 ▸). These indicate that cocrystallization is an effective approach for enhancing the physicochemical properties of APIs.

Coumarins, also known as benzopyrones, are present in considerable concentrations in plants. They have also been reported from microbes and animal sources. Coumarin derivatives reported in various natural and synthetic compounds are known for a range of pharmacological properties including anti-inflammation, anti-oxidant, anti-leishmanial, anti-cancer, anti-HIV, anti-microbial and antiviral effects (Beillerot *et al.*, 2008 ▸; Wu *et al.*, 2009 ▸; Matos *et al.*, 2011 ▸; Cuellar *et al.*, 2022 ▸). Coumarin-3-carboxylic acid (**CU**, 2-oxobenzopyrane-3-carboxylic acid) is a synthetic coumarin analogue (Stuart, 1886 ▸). Naturally occurring coumarins, including warfarin, herniarin and aesculetin, have various biological and therapeutic properties (Lacy & O’Kennedy, 2004 ▸; Borges *et al.*, 2005 ▸; Ahmad & Misra, 1997 ▸). Furthermore, coumarin derivatives exhibit optical properties and are widely used in laser dyes, solar cells and florescent probes (Skowronski *et al.*, 2015 ▸; Tasior *et al.*, 2015 ▸; Jones & Rahman, 1994 ▸; Hara *et al.*, 2003 ▸). The literature reports that 7-diethylamino-coumarin-3-carboxylic acid has been utilized as a laser dye, fluorescent label and biomedical inhibitor (Wu *et al.*, 2019 ▸). The cocrystallization of **CU** with various coformers was reported to modify its luminescence properties (Yan *et al.*, 2012 ▸). Recently, our research group has also reported cocrystallization of **CU** with thiourea to study its change as an antioxidant agent (Shahbaz *et al.*, 2022 ▸).

**Figure 1:**
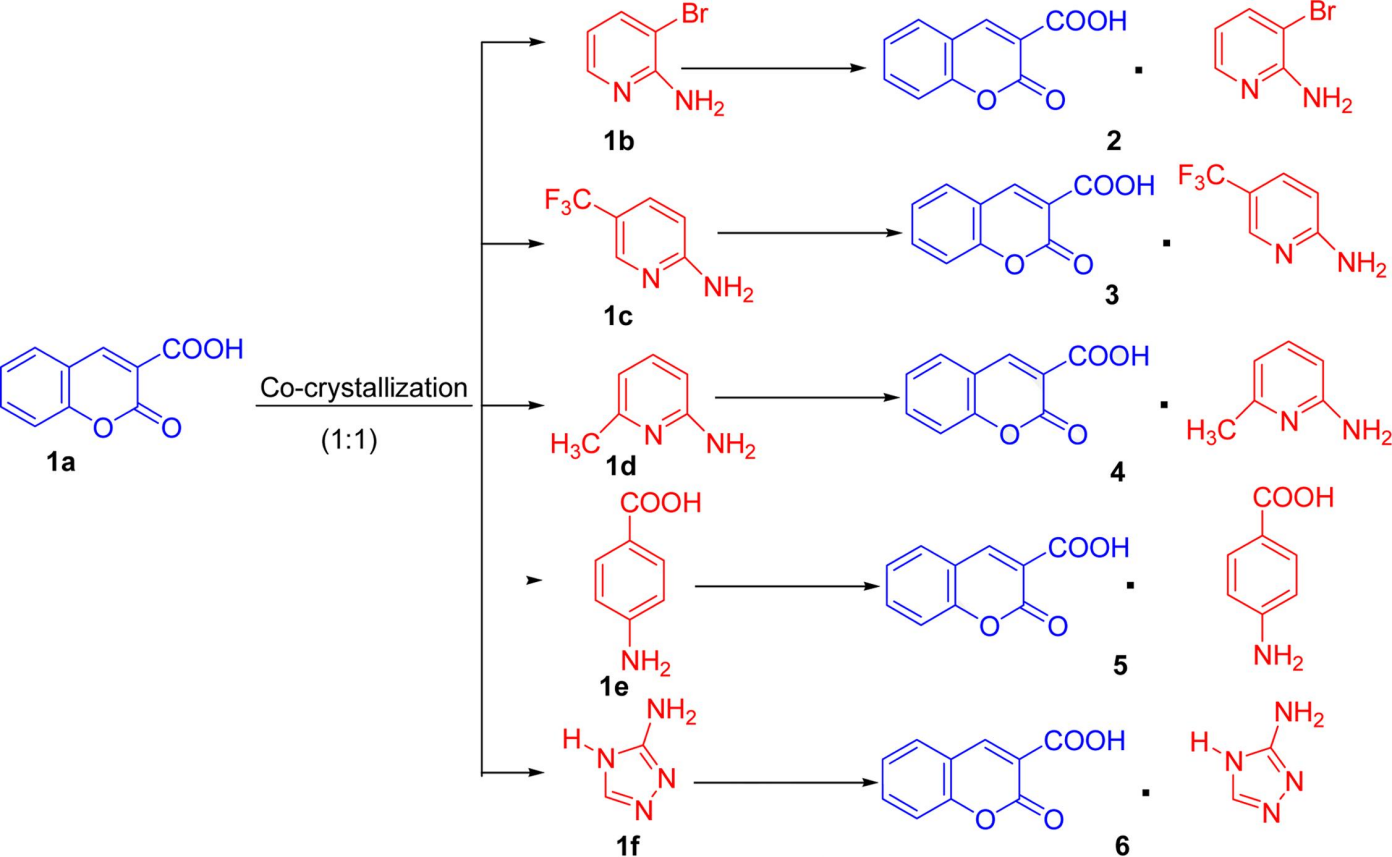
Schematic representation of the synthesis of cocrystals **2**–**6** by neat grinding in a mixer mill.

Here we focused on the cocrystallization of **CU** with various coformers, such as 2-amino-3-bromo­pyridine (**1b**), 2-amino-5-(tri­fluoro­methyl)-pyridine (**1c**), 2-amino-6-methyl­pyridine (**1d**), *p*-amino­benzoic acid (**1e**) and amitrole (**1f**) (Fig. 1 ▸). The selection of coformers was based on their amino functionalities, *i.e.* their ability to form hetrosynthons (Thalladi *et al.*, 1996 ▸) with **CU** (**1a**, which contains two carbonyls) via O—H⋯O and N—H⋯O bonding. The *in vitro* anti-leishmanial activity against the promastigotes miltefosine (MIL)-resistant *Leishmania tropica* (*L. tropica*) – the causative agent of CL – and cytotoxicity against the 3T3 (normal fibroblast) cell line of synthesized cocrystals were evaluated, and the results showed an improved anti-leishmanial potential in cocrystals compared with the pure API. The synthesized cocrystals were characterized using various solid-state characterization techniques including single-crystal X-ray diffraction (SCXRD), powder X-ray diffraction (PXRD) and Fourier transform infrared (FTIR) spectroscopy, followed by a study of the thermal stability via differential scanning calorimetry (DSC) and thermogravimetric analysis (TGA).

## 2. Experimental

### 2.1. Materials

Coumarin-3-carb­oxy­lic acid (99%) (**1a**), 2-amino-3-bromo­pyridine (97%) (**1b**), 2-amino-5-(tri­fluoro­methyl)-pyridine (97%) (**1c**), 2-amino-6-methyl­pyridine (97%) (**1d**), *p*-amino­benzoic acid (97%) (**1e**) and amitrole (97%) (**1f**) were purchased from Sigma–Aldrich (Germany). HPLC-grade solvents were used without further purification.

### 2.2. Synthesis of cocrystals

**CU** (**1a**) (88.0 mg, 0.46 mmol) was cocrystallized with the coformers **1b** (79.5 mg, 0.46 mmol), **1c** (49.7 mg, 0.46 mmol), **1d** (74.5 mg, 0.46 mmol) **1e** (63.4 mg; 0.46 mmol) and **1f** (38.60 mg, 0.46 mmol) (coformers) in a 1:1 stoichiometric ratio by neat grinding in a mixer mill (MM400, Germany) for 90 min at 30 s^−1^ (Fig. 1 ▸). The grinded material obtained was dissolved in various solvents: cocrystals **2**, **4** and **5** in hot aceto­nitrile (70°C), and cocrystals **3** and **6** in methanol (65°C), and the solutions were maintained for crystallization at room temperature for 4 to 5 days. In addition to the above-mentioned coformers, we attempted to cocrystallize **CU** (**1a**) with a range of other available coformers (Table S2) in a 1:1 stoichiometric ratio via neat grinding in a mixer mill (MM400, Germany) for 30–120 min at 30 s^−1^, but we were unsuccessful.

### 2.3. Single-crystal X-ray diffraction

SCXRD analyses of all single-crystals were carried out on a Bruker D8 venture (Germany), fitted with a photon detector with CMOS 100 technology. The crystals were irradiated by graphite-monochromated Cu *K*α radiation (λ = 1.54178 Å) at 100 (2) to 300 (2) K. Integration and reduction of data were completed using the Bruker *SAINT* software (Bruker, 2016 ▸). The structures were solved by direct methods and Fourier transformation techniques using the *SHELXL* program (Sheldrick, 2015 ▸). Structures were refined by full-matrix least-squares calculations on *F* ^2^. All non-hydrogen atoms were refined with anisotropic displacement parameters and placed at geometrically idealized positions, and all hydrogen atoms were located by difference maps and refined isotropically. The inter-molecular interactions between the molecules were calculated using *PLATON* (Spek, 2003 ▸). The crystal-packing diagrams and 3D structures were drawn using *Mercury* (Macrae *et al.*, 2008 ▸) and *ORTEP* (Farrugia, 1997 ▸). Crystallographic and refinement data are summarized in Table 1 ▸.

### 2.4. Powder X-ray diffraction

The bulk samples of all synthesized cocrystals were characterized via PXRD analysis on a Bruker D8 Advance diffractometer equipped with a LynxEye detector and monochromatic Cu *K*α radiation (λ = 1.54060 Å) sources at 25°. The powdered samples were placed in an acrylic sample holder. The data were collected initially within the range 5 to 65° (2θ) with a step size of 0.036°. In order to determine the full structure, a continuous scan mode was used.

### 2.5. Hirshfeld surface analysis

Hirshfeld surfaces and 2D fingerprint plots were generated with *Crystal Explorer* (version 17.5; Spackman & Jayatilaka, 2009 ▸; Mackenzie *et al.*, 2017 ▸) using the automatic procedures implemented in the software. These surfaces were mapped with a normalized contact distance (*d*_norm_), shape-index, curvedness and *ab initio* electrostatics surface parameters, with automatic values.

### 2.6. Fourier transform infrared studies

FTIR spectra were recorded for **CU**, the coformers and its cocrystals on a Bruker Vector 22 FTIR spectrometer (Germany). All samples were analyzed via the KBR disk technique, and a spectrum was collected under identical conditions, the spectrum scan range was 400 to 4000 cm^−1^ with a resolution of 2 cm^−1^ and an accumulation of 10 scans.

### 2.7. Thermal analysis

DSC and TGA were performed on a LINSEIS STA PT1600 with heating rate of 10°C min^−1^. About 20–25 mg of samples were crimped in a ceramic pan and scanned from 30 to 600°C under dry N_2_ gas purging. The *Linseis TA* software (version 1.0; Linseis Messgeraete GmbH) was employed for collecting data.

### 2.8. Biological screening

*In vitro* biological activities of **CU**, coformers and synthesized cocrystals **2**–**6** were evaluated for their MIL-resistant *L. tropica* promastigotes and cytotoxicity against T3 normal mouse fibroblast cell line. Detailed methodologies of the biological assays are provided in the supporting information.

### 2.9. Statistical analysis

Three replicates were used in each experiment, unless otherwise stated. All results were presented as mean standard deviations. A one-way ANOVA was used to analyze statistical differences at *P* < 0.05 (95% confidence interval) in conjugation with Tukey’s Multiple Comparison Test using the *Graph Pad Prism* software (version 5; California, USA; https://www.graphpad.com).

## 3. Results and discussion

### 3.1. Selection of coformers

Based on the literature review, functional groups capable of forming supramolecular synthons via hydrogen bonds such as acid⋯acid (COOH⋯COOH) and acid⋯amino (COOH⋯NH_2_) are the essential structural features known to facilitate the formation of cocrystals (Nugrahani & Jessica, 2021 ▸; Desiraju, 1995 ▸). The coformers in the present study evidenced the above statement as **1b**, **1c**, **1d** and **1f** possess an amino-pyridine functionality and are well known for forming dimeric hetrosynthons in crystal structures. On the other hand, coformer **1e** showed the carb­oxy­lic acid homosynthons motif. Moreover, the present study revealed that all the coformers (**1b**–**1f**) demonstrate non-cytotoxicity against the normal 3T3 fibroblast cell line. Therefore, it was considered worthwhile to explore the potential of conformers not only as supramolecular synthons, but also as coformers of bioactive cocrystals.

### 3.2. Single-crystal X-ray diffraction analysis

**Figure 2:**
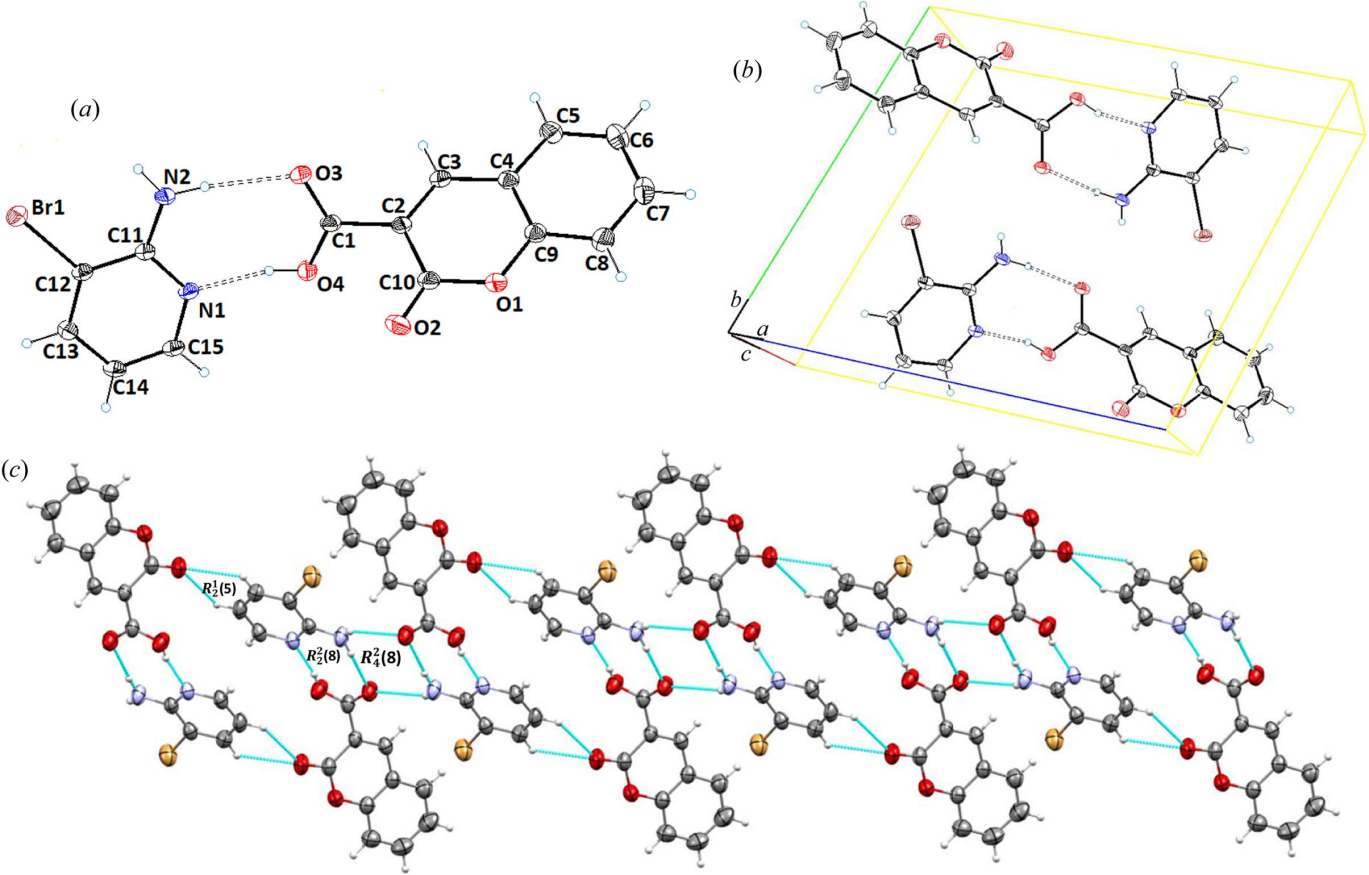
(*a*) *ORTEP* view; (*b*) unit-cell packing; and (*c*) hydrogen-bonded framework of **CU**:**1b** (1:1), cocrystal **2**.

SCXRD revealed that cocrystal **2** (**CU**:**1b**) crystallizes in the triclinic space group *P* 1 and contains one molecule each of **CU** (**1a**) and **1b** in the asymmetric unit [Fig. 2 ▸(*a*)]. Structural analysis revealed that the **CU** (**1a**) molecule was composed of a planar coumarin ring (O1/O2/C2–C10) with a carb­oxy­lic acid functionality at C2. Structurally, the coformer consists of –NH_2_ (C11) and a –Br (C12) planar pyridine ring (C11–C15/N1). Molecular planarity of **CU** (**1a**) in cocrystal **2** was achieved to a maximum deviation of 0.009 Å from the root-mean-square (r.m.s.) plane for C4. The dihedral angle of the carb­oxy­lic functionality to the planar coumarin ring in cocrystal **2** is 51.61°. The torsion angle along O4—C1—C2—C10 was found to be −53.5 (4)° [Fig. 2 ▸(*a*)]. In the crystal structure, both carbonyls of lactone and the acid of the API (**CU**) are involved in the hydrogen bonding with **1b**, and therefore contribute towards cocrystal stabilization. The coformer **1b** interacts with the neighboring **CU** molecule via N2—H2A—O3, N2—H2B⋯O3, O4—H4⋯N1, C13—H13⋯O2 and C15—H15⋯O4 inter-molecular interactions [Fig. 2 ▸(*b*)] to form dimeric 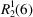 and 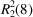 , and tetrameric 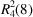 ring motifs (Etter, 1990 ▸) [Fig. 2 ▸(*c*)]. The key hydrogen-bonding interactions are presented in Table 2 ▸.

**Figure 3:**
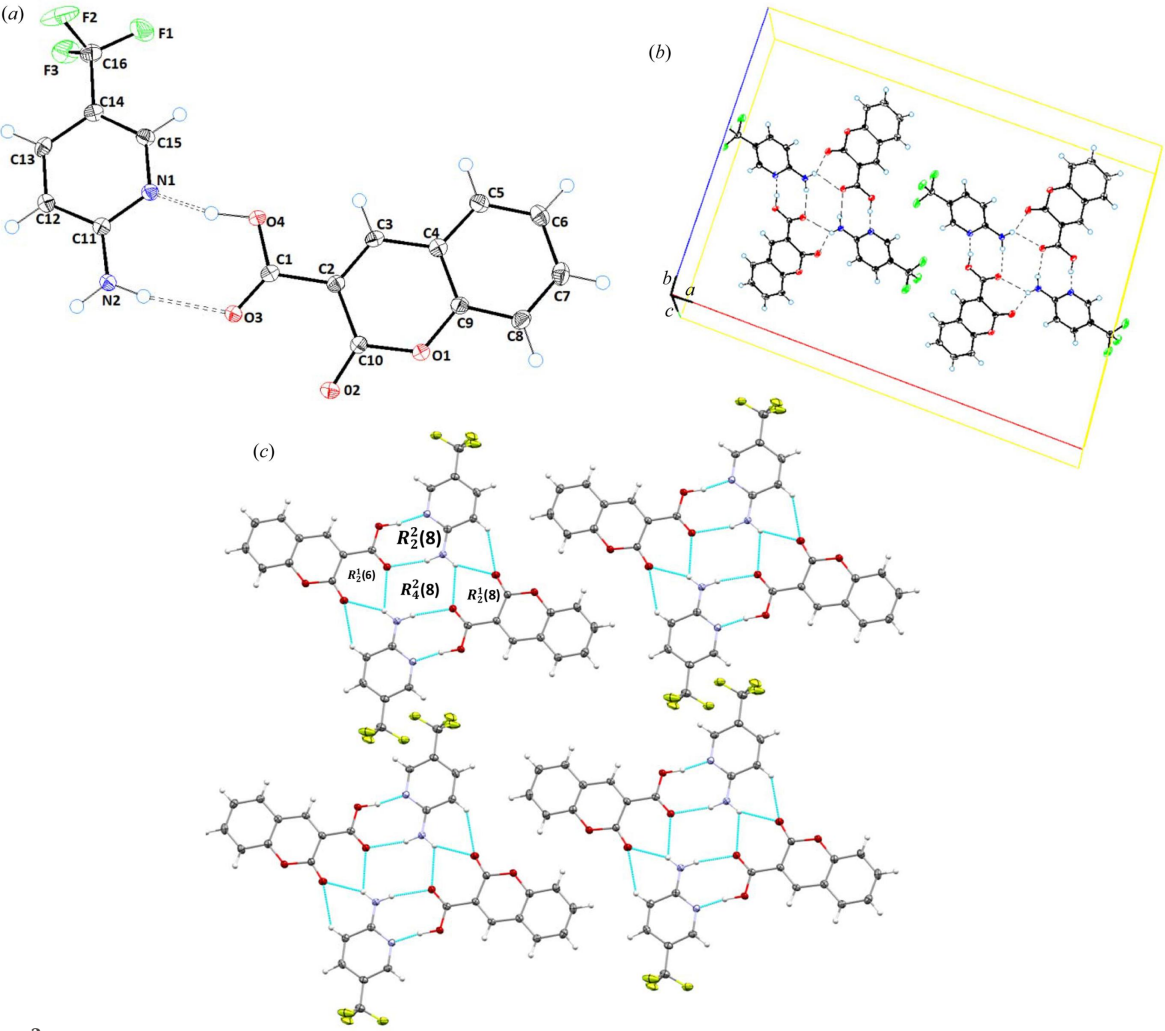
(*a*) *ORTEP* view; (*b*) unit-cell packing; and (*c*) hydrogen-bonded framework of **CU**:**1c** (1:1), cocrystal **3**.

Cocrystal **3** (**CU**:**1c**) crystallizes in the monoclinic space group *C*2/*c* in a 1:1 stoichiometric ratio, *i.e.* the asymmetric unit comprises a molecule of **CU** (**1a**) and a molecule of **1c** [Fig. 3 ▸(*a*)]. The planarity of **CU** in cocrystal **3** has a maximum deviation of 0.006 Å for atom C4 from the best root-mean-square plane of the coumarin ring. In the crystal structure of cocrystal **3**, the **1c** moiety is linked with three **CU** molecules via classical N2—H2B⋯O2, N2—H2B⋯O3 and N2—H2A⋯O3, and non-classical C12—H12⋯O2 and C8—H8⋯O1 hydrogen bonds with donor–acceptor distances of 2.9669 (16), 2.8755 (14), 2.9292 (15), 3.5228 (16) and 3.2087 (16) Å, respectively [Fig. 3 ▸(*b*)]. These hydrogen bonds form dimeric 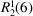 and 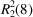 , and tetrameric 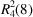 loop graph set ring motifs [Fig. 3 ▸(*c*), Table 2 ▸].

**Figure 4:**
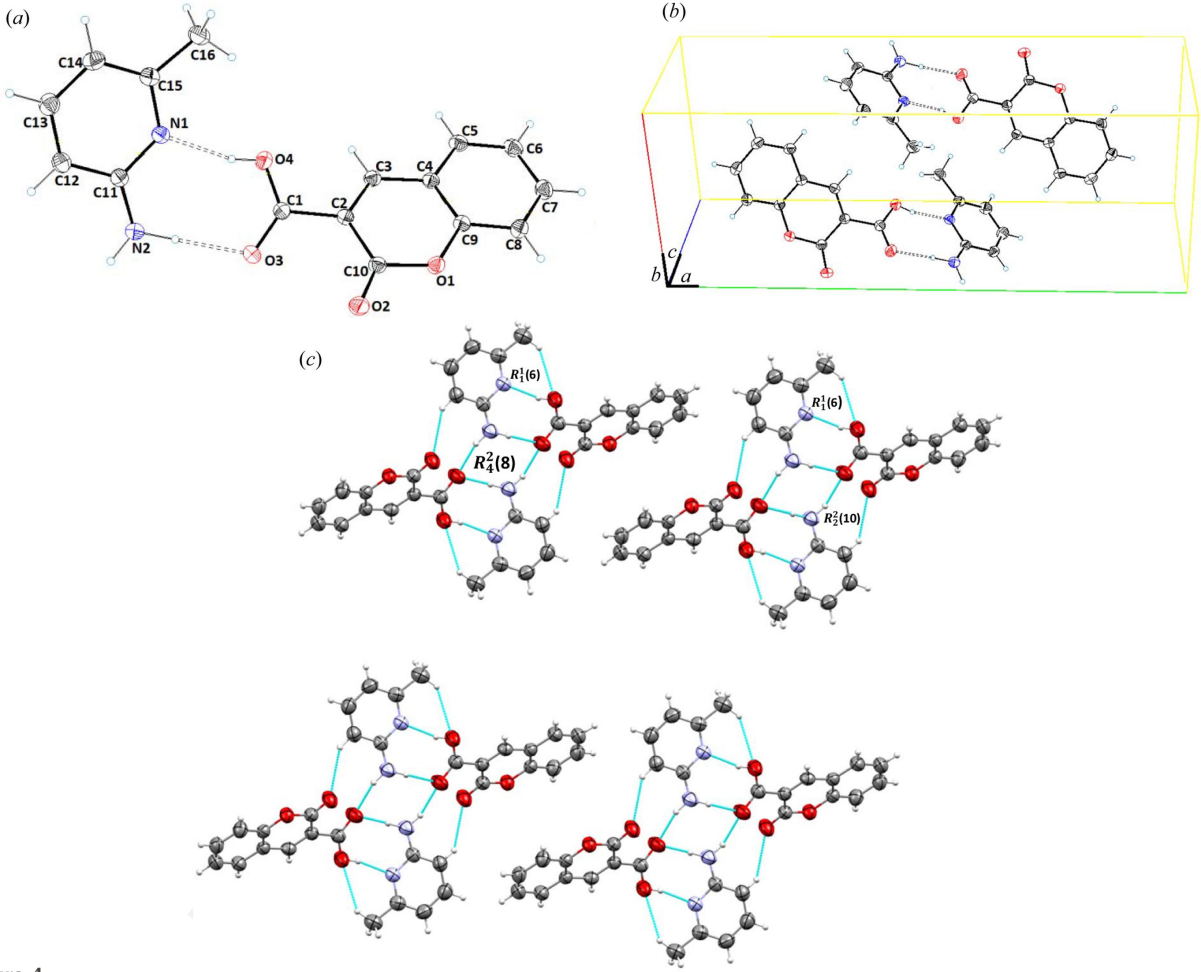
(*a*) *ORTEP* view; (*b*) unit-cell packing; and (*c*) 2D hydrogen-bonded framework of **CU**:**1d** (1:1), cocrystal **4**.

Cocrystal **4** (**CU**:**1d**) crystallizes in the monoclinic space group *P*2_1_/*c*. The asymmetric unit contained one molecule each of **CU** and **1d** [Fig. 4 ▸(*a*)]. The structure parameters of the API (**CU**) in cocrystal **4** were found to be similar to cocrystals **2** and **3**, *i.e.* a maximum root-mean-square deviation of 0.006 Å for C4. The carb­oxy­lic acid was found to be inclined at an angle of 33.63° along the planar coumarine ring. In the crystal structure, **CU** and **1d** moieties interact through inter-molecular hydrogen N2—H2A⋯O4, O3—H3A⋯N1, C12—H12⋯O2, C16—H16B⋯O4 and C16—H16A⋯O3 bonds with donor–acceptor distances of 2.817 (3), 2.658 (2), 3.425 (3), 3.533 (3) and 3.410 (3) Å, respectively [Fig. 4 ▸(*b*)]. These hydrogens bonds form dimeric and tetrameric 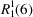 , 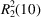 and 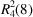 ring motifs [Fig. 4 ▸(*c*), Table 2 ▸].

**Figure 5:**
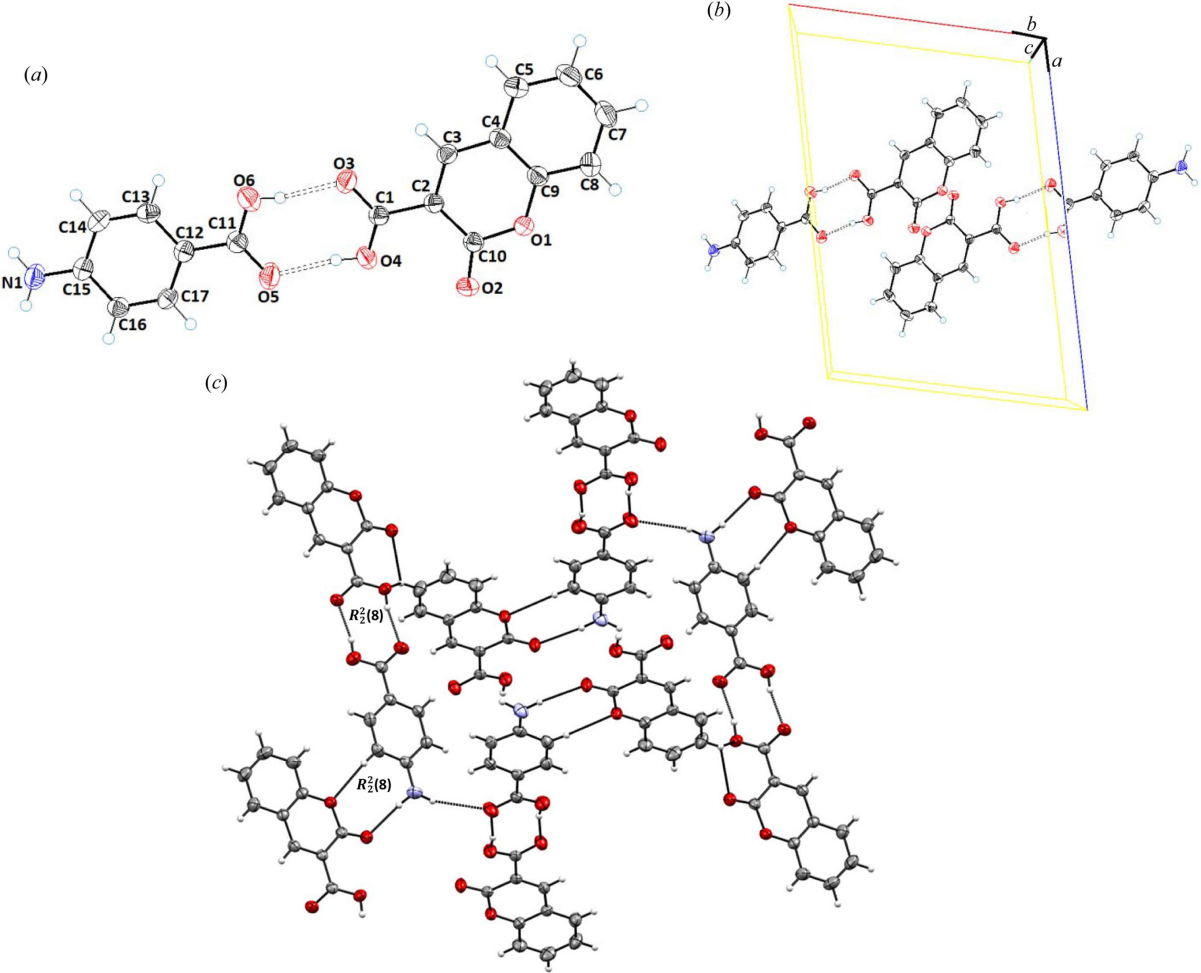
(*a*) *ORTEP* view; (*b*) unit-cell packing; and (*c*) hydrogen-bonded framework of **CU**:**1e** (1:1), cocrystal **5**.

Cocrystal **5** (**CU**:**1e**) also crystallizes in the monoclinic space group *P*2_1_/*n* with the asymmetric unit consisting of a molecule each of **CU** and **1e**, as shown in Fig. 5 ▸(*a*). In the crystal structure of cocrystal **5**, the structural features of the **CU** molecule were found to be similar to cocrystal **2**, whereas **1e** was found to consist of a benzene ring (C12–C17) substituted with carb­oxy­lic acid and amino groups at C12 and C15, respectively. In cocrystal **5**, the deviation of 0.004 Å of the C4 atom was observed for the planar coumarin ring (O1/C2–C10) from the root-mean-plane. In the crystal structure, the carbonyl (–C=O), hydroxyl (–OH) and amino (–NH_2_) groups of **CU** and **1e** contribute towards the stability of the cocrystal via O4—H4A⋯O6, O5—H5A⋯O3, N1—H1B⋯O6 and N1—H1A⋯O2 hydrogen bonds with donor–acceptor distances of 2.6581 (14), 2.6417 (14), 3.4143 (17) and 3.1762 (16) Å, respectively [Fig. 5 ▸(*b*)]. The network was further extended via non-classical C16—H16⋯O1 hydrogen bonds with a donor–acceptor distance of 3.4789 (16) Å. These interactions form 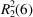 and 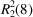 graph set ring motifs (Etter, 1990 ▸) as depicted in Fig. 5 ▸(*c*) and Table 2 ▸.

**Figure 6:**
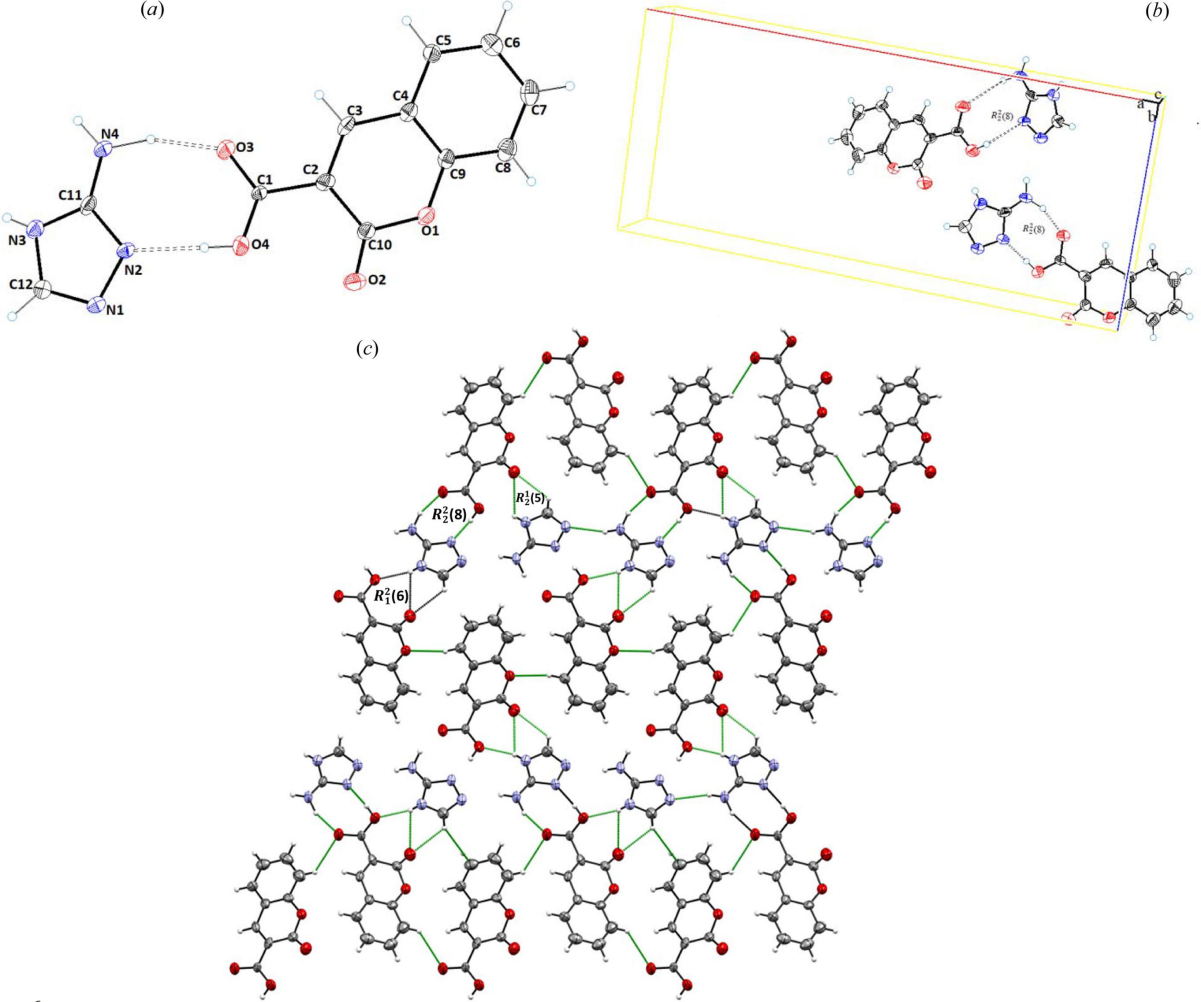
(*a*) *ORTEP* view; (*b*) unit-cell packing; and (*c*) hydrogen-bonded framework of **CU**:**1f** (1:1), cocrystal **6**.

Cocrystal **6** (**CU**:**1f**, 1:1) crystallizes in the orthorhombic space group *Pna*2 [Fig. 6 ▸(*a*)]. **CU** within the structure was found to be similar to observations in the previously described cocrystals, whereas the **1f** coformer (N1–N4/C11–C12) exhibited a planar triazole ring (N1/N2/N3/C11/C12) substituted with an amino group at C11. In cocrystal **6**, the O4—H4⋯N2, N4—H4A⋯O3, N4—H4B⋯N1, N3—H3A⋯O2 and N3—H3A⋯O4 interactions form 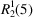 , 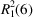 and 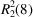 ring motifs. The cocrystal also possesses the non-classical hydrogen bond C8—H8⋯O3 with a donor–accepter distance of 3.334 (12) Å, which contributes to its molecular stability [Fig. 6 ▸(*c*), Table 2 ▸].

### 3.3. Hirshfeld surface analysis

The Hirshfeld surface analysis was used to quantify the nature, regions and types of inter-molecular interactions in the crystal structure via mapping their properties in various modes, such as *d*_norm_, shape-index, curvedness, electrostatic potential surface and 2D plots. The dark-red and blue regions indicate the shorter (close contacts) and longer (distant contacts) distances in comparison with the van der Waals radii, respectively, and the white regions reflect a distance equal to the sum of the van der Waals radii (Venkatesan *et al.*, 2016 ▸). The darkest red spots on the Hirshfeld surface exhibit the O—H⋯O, O—H⋯N and N—H⋯O contacts. These strong inter-molecular interactions facilitate the formation of cocrystals (Fig. S1). The Hirshfeld surfaces, mapped over the shape-index and curvedness surface, are depicted in Figs. S2 and S3. These surfaces were used to present weak inter-molecular interactions in the cocrystal and the overall packing in the crystal structure. The presence of blue and red triangles on shape-index surfaces and flat green regions on the curvedness indicate the C—H⋯π or π-stacking in cocrystals. Another Hirshfeld surface was mapped over the calculated *ab initio* electrostatic potential on the Hartree–Fock (HF) level of theory using the 6-311G(d.p) basis set. Fig. S4 shows that the positive electrostatic potential over the surface are hydrogen-bond donors (blue regions) and the negative electrostatic potential are the hydrogen-bond acceptors (red regions).

**Figure 7:**
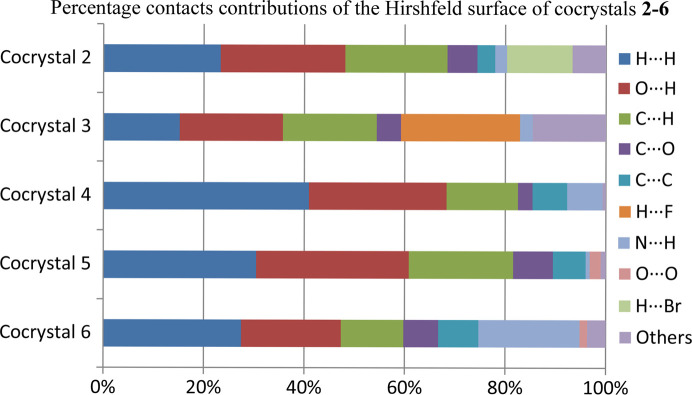
Bar plot representing the 2D fingerprint plots, showing the percentage contributions of the contents of cocrystals **2**–**6**.

The overall 2D fingerprint plots resolved into all types of contacts (H⋯H, O⋯H, C⋯H, C⋯N, N⋯H, C⋯O, C⋯C, H⋯F, F⋯F, H⋯N, O⋯O, Br⋯H, Br⋯C and N⋯O) and their relative percentage populations are shown in the bar graph (presented in Fig. 7 ▸). The main contacts (H⋯H, O⋯H, N⋯H and H⋯F) are the major contributors towards the formation of the Hirshfeld surface. The O⋯H interactions are depicted in Figs. S5–S9 by inside sharp spikes, the N⋯H contacts are revealed by sharp edge spikes and H⋯H contacts are indicated by the main body of the fingerprint plots. The O⋯H interactions make up the largest proportion, indicating that they are the main contributors to the stabilization of cocrystals **2**–**6**.

### 3.4. Powder X-ray diffraction analysis

**Figure 8:**
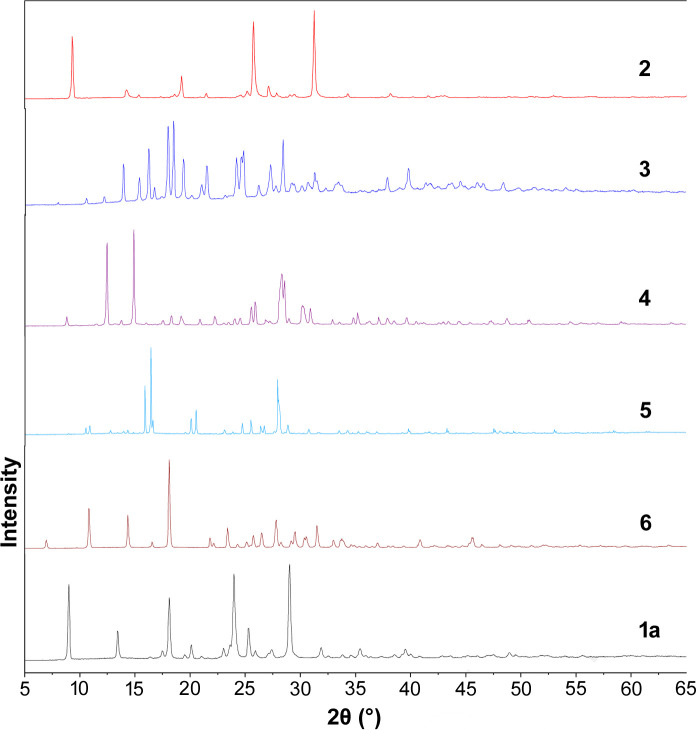
PXRD Patterns of **CU** (**1a**) and the synthesized cocrystals **2**–**6**.

The PXRD data further supported the successful synthesis of the cocrystals. The overlay of PXRD patterns of individual component such as **CU** (**1a**) with PXRD patterns of the synthesized cocrystals **2**–**6** obtained from the slow evaporation method indicate that the new crystalline phase has a unique diffraction pattern and is different from the individual components (Fig. 8 ▸). The diffraction pattern of intact **CU** (**1a**) shows that the solid is a highly crystalline powder with sharp diffraction peaks at 2θ = 9.02, 13.45, 18.13, 13.93, 25.27 and 28.97°. Cocrystal **2** shows the diffraction peaks at 2θ = 8.94, 18.06, 24.01 and 29.05°. The characteristic peaks of cocrystal **3** appeared at 2θ = 13.92, 16.24, 17.95, 18.45, 19.40, 21.50, 24.15 and 28.43°. Moreover, the characteristic peaks of cocrystal **4** are 2θ = 11.20, 13.23, 22.05, 22.41, 24.73 and 26.01°. Cocrystals **5** and **6** revealed characteristic diffraction peaks at 2θ = 15.88, 16.42, 20.09, 20.52 and 27.92°; and 2θ = 10.37, 13.56, 17.01, 21.86, 25.93 and 29.30°, respectively.

### 3.5. FTIR analysis

FTIR analyses of **CU** (**1a**), **1b**, **1c**, **1d**, **1e**, **1f** and their cocrystals **2**–**6** were performed and are presented in the supporting information (Figs. S10–S14).

In cocrystal **2** (**CU**:**1b**), the –NH_2_ stretching vibrations appear to be red-shifted at 3363 cm^−1^ compared with 3459 cm^−1^ observed for **1b**. **CU** (**1a**) revealed a stretching C=O bond of the lactone carbonyl at 1745 cm^−1^, whereas the red-shifted absorption band appeared at 1723 cm^−1^ in cocrystal **2**. The slight red-shift in the C=O bond of the acid carbonyl from 1683 to 1680 cm^−1^ in the cocrystal and blue-shift in C—O from 1225 to 1250 cm^−1^ clearly support the involvement of hydrogen bonding in the formation of cocrystal **2** (Fig. S10). Similarly, in cocrystal **3** (**CU**:**1c**), the –NH_2_-stretching vibration appeared as a strong band at 3386 cm^−1^ which was found to be red-shifted in comparison with the –NH_2_ stretching vibration (3504 cm^−1^) in **1c**. Similarly, in cocrystal **3**, a strong absorption band at 1759 cm^−1^ appeared due to the C=O of the lactone moiety and showed blue-shifting from 1745 cm^−1^, the stretching frequency of the lactone carbonyl in **CU**. In addition, the blue-shift in C—F from 1329 to 1334 cm^−1^ indicates the involvement of the –CF_3_ functionality of the coformer in hydrogen bonding (Fig. S11). In cocrystal **4** (**CU**:**1d**), the C=O (lactone carbonyl) absorption band appeared at 1731 cm^−1^ and revealed a red-shift compared with the stretching frequency observed for **CU** (1745 cm^−1^). The blue-shifted olefinic C=C bond-stretching frequency from 1610 to 1625 cm^−1^ and red-shift in stretching frequency of C=O (acid carbonyl) from 1683 to 1663 cm^−1^ further support the involvement of the carb­oxy­lic acid moiety of **CU** in hydrogen bonding with conformer **1d**. Furthermore, the broadening of –NH_2_ and carb­oxy­lic –OH absorption bands (3500 to 2756 cm^−1^) in cocrystal **4** indicates strong hydrogen bonding between the acid and amine groups (Fig. S12). In the IR spectrum of cocrystal **5** (**CU**:**1e**), the stretching frequencies signify that both the –COOH group of **CU** and **1e** do not get deprotonated. The IR spectra showed three intense bands at 1748, 1675 and 1632 cm^−1^. The strong band at 1675 cm^−1^ of the cocrystal may be due to overlapping of the acid carbonyl group of **CU** (1683 cm^−1^) and that of coformer **1e** (1667 cm^−1^). In **1e**, stretching of the N–H hydrogen bond at 3462 cm^−1^ was observed, whereas in the cocrystal the N–H stretching vibration was observed at 3460 cm^−1^ with a red-shift (Fig. S13). In cocrystal **6** (**CU**:**1f**) the C=O carbonyl absorption band of lactone appeared at 1738 cm^−1^, whereas in **CU** it appears at 1745 cm^−1^, this red-shift indicates the involvement of C=O (lactone carbonyl) in hydrogen bonding with the **1f** coformer. The C=O of the acid carbonyl from 1683 to 1703 cm^−1^ was attributed to red- and blue-shifts in the olefinic bond-stretching frequency from 1610 to 1613 cm^−1^. The –NH_2_ stretching was observed as a sharp band at 3431 cm^−1^ in **1f**, whereas in cocrystal **6** the –NH_2_ stretching was observed with a blue shift at 3330 cm^−1^ (Fig. S14). In conclusion, the red and blue shifts of the characteristic functional group stretching frequencies in the IR spectra of synthesized cocrystals **2**–**6** clearly demonstrate the role of hydrogen bonding in cocrystallization.

### 3.6. Thermal analysis

To analyze the thermal behavior of newly synthesized cocrystals, DSC and TGA measurements were performed. The thermal properties of the synthesized cocrystals were significantly different from those of the pure APIs and coformers. DSC and TGA thermograms of pure **CU** (**1a**), coformers **1b**–**1f** and their synthesized cocrystals **2**–**6** are presented in the supporting information (Fig. S15–S19). The DSC curve of **CU** (**1a**) revealed a eutectic endotherm at 191.78°C whereas **1b** revealed two endotherm peaks at 67.29 and 199.8°C. However, the DSC spectra of cocrystal **2** exhibited a small endotherm at 113.6°C, followed by a second larger endotherm at 189.1°C, indicating the development of a new solid phase. The TGA analysis of cocrystal **2** showed it is thermally stable up to 113.8°C, followed by a percentage mass loss of 7.2% with a temperature increase up to 189.1°C; 99.9% mass loss occured at 491.8°C, compared with **CU** and **1b**, which showed thermal stability up to 191.78 and 67.29°C, respectively (Fig. S15). The DSC spectrum of cocrystal **3** was found to have an exothermic peak at 160.4°C, whereas a sharp endotherm appeared at 180.04°C, distinctly different from **CU** (eutectic melting endotherm at 191.78°C). The coformer **1c** exhibited two sharp endotherms at 48.38 and 175.56°C, demonstrating the new crystalline phase, *i.e.* cocrystal **3**. The TGA profile of cocrystal **3** exhibited a thermal stability up to 160.4°C with a percentage mass loss of 17.3% and complete mass loss of 99.68% with an increased temperature up to 250.10°C. The results indicate that the synthesized cocrystal was stable up to 160.4°C, compared with **CU** (191.78°C) and **1c** (48.38°C) (Fig. S16). The DSC spectrum of cocrystal **4** exhibited a eutectic endotherm at 178.73°C, which differed from pure **CU** (191.78°C), demonstrating the new cocrystal phase. TGA of cocrystal **4** revealed thermal stability up to 178.73°C with a mass loss of 18.15% and with complete mass loss of 99.89% with an increased temperature up to 310.30°C (Fig. S17). The DSC thermogram of cocrystal **5** showed an endothermic peak at 170.5°C, different from **CU** (191.78°C) and **1e** (186.0°C). This clearly indicates the development of a new solid state (*i.e.* cocrystal **5**), which was found to be stable up to 170.5°C with a mass loss of 7.20%. The TGA curves of synthesized cocrystal **5** revealed noticeable changes in the thermal decomposition pattern (Fig. S18). The DSC spectra of cocrystal **6** exhibited an endotherm peak at 149.0°C which indicates the development of a new crystalline phase. Moreover, TGA of cocrystal **6** revealed that it is thermally stable up to 123.58°C, with a loss of 5.9%. In cocrystal **6**, we noted that, on the basis of TGA curve analysis, the decomposition of the cocrystal appears to pass through three stages, although further work is required to better understand this mechanism (Fig. S19).

### 3.7. MIL-resistant *L. tropica*

The alkyl­phospho­choline drug MIL is a broad-spectrum drug that is active against various parasitic species, cancer cells, as well as against a number of pathogenic fungi and bacteria (Dorlo *et al.*, 2012 ▸). Knowledge about MIL resistance in *L. tropica* is limited to defects in drug internalization (defected inner translocation of MIL) and increased drug efflux (Pérez-Victoria *et al.*, 2006 ▸). According to Hendrickx *et al.* (2014 ▸, 2012 ▸), when emergence of any degree of resistance occurs in the MIL-resistant culture, the resistance does not revert back to wild-type (WT) phenotype, despite the removal of MIL-selective pressure.

**Figure 9:**
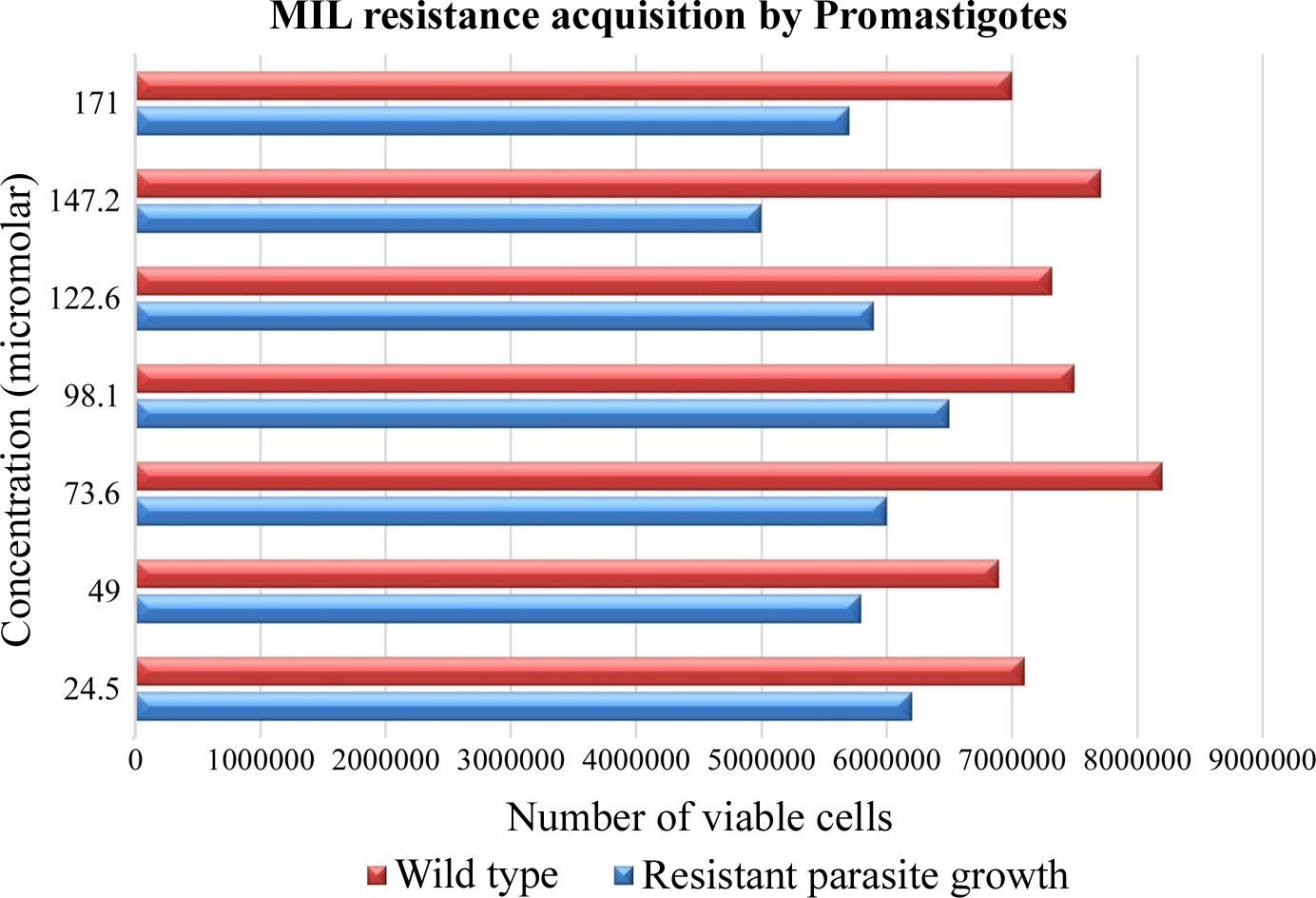
MIL-resistant *L. tropica* line generated by step-wise selection. 1 × 10^6^ log-phase promastigotes were incubated in the presence of a range of drug concentrations. The surviving cells were quantified with trypan blue dye. Populations of parasites were grown in increasing concentrations of MIL, showing increased resistance to MIL. Bars of both resistant and WT parasites represent the more or less similar growth patterns.

*L. tropica* MIL-unresponsive/resistant parasites were developed and maintained using a step-wise selection of the drug MIL up to a concentration of 196 µ*M*. No significant differences in growth patterns were observed between WT- and MIL-resistant strains. Fig. 9 ▸ shows that no inhibitory effects of MIL on cell proliferation of *L. tropica* were observed, demonstrating successful emergence of resistance via a dose-dependent increase of MIL.

A potential disadvantage in the use of MIL in leishmanial assay is the emergence of *in vitro* drug resistance (Varela-M *et al.*, 2012 ▸). Furthermore, this drug is found to be potentially teratogenic, and is not recommended for pregnant women (Committee, 2010 ▸; Murray *et al.*, 2005 ▸). Mechanisms that are responsible for the resistance acquisition in the *L. tropica* parasite against MIL include reduction in drug uptake, increased efflux and alteration in permeability of the plasma membrane (Pérez-Victoria *et al.*, 2003 ▸; Seifert *et al.*, 2003 ▸; Kulshrestha *et al.*, 2014 ▸; Sánchez-Cañete *et al.*, 2009 ▸; Mondelaers *et al.*, 2016 ▸).

Hence it is a necessity to find alternative therapeutic options for leishmaniasis. During the current study, the resistant strain was generated and a series of cocrystals were evaluated against the parasitic line.

### 3.8. Inhibitory potential of the synthesized cocrystal against MIL-resistant *L. tropica in vitro*

Understanding the role of mixtures of molecules in any bio-system is very complicated and difficult to understand. The synergistic effect on biological activities due to a multi molecular system is well reported in the literature, perhaps the best explanation for biological activities of plant extracts (the complex mixture of natural products). The co-crystals are systematically designed multi-component molecules in a stoichiometric ratio. Therefore, the orientation of cocrystal components due to hydrogen bonding is responsible for the change in physicochemical and biological properties compared with the individual components. The present study demonstrates the susceptibility of a synthesized series of cocrystals (**2**–**6**) of **CU** (**1a**) with coformers (**1b** and **1c**) against the MIL-resistant *L. tropica.* Cocrystal **CU**:**1d** (**4**) appeared as a potent (<0.05) anti-leishmanial agent against resistant promastigotes with a IC_50_ value of 48.71 ± 0.75 µ*M* against the tested standard drug (IC_50_ = 169.55 ± 0.078 µ*M*). Cocrystal **CU**:**1b** (**2**) appeared to be the second most potent (<0.05) anti-leishmanial agent with a IC_50_ value of 61.83 ± 0.59 µ*M*, followed by cocrystal **CU**:**1c** (**3**, IC_50_ = 125.7 ± 1.15 µ*M*). Among all coformers, only **1f** showed potent anti-leishmanial effects (IC_50_ = 78.0 ± 0.096 µ*M*); however, the synthesized cocrystal of **CU** with **1f** (**6**) appeared to be inactive and therefore demonstrated the role of supramolecular features in the modification of the orientation of the API and coformer and finally the molecular properties in a way to make the molecule inactive. All 1:1 physical mixtures of APIs and coformers also appeared to be inactive. In the case of mixtures, the reason for the complete loss of anti-leishmanial activity cannot be explained clearly; however, both the role of concentration and the free dispersion of **CU** and coformers (**1b**–**1e**) in the system could be possible reasons (Table 3 ▸).

### 3.9. *In vitro* cytotoxicity evaluation

**Figure 10:**
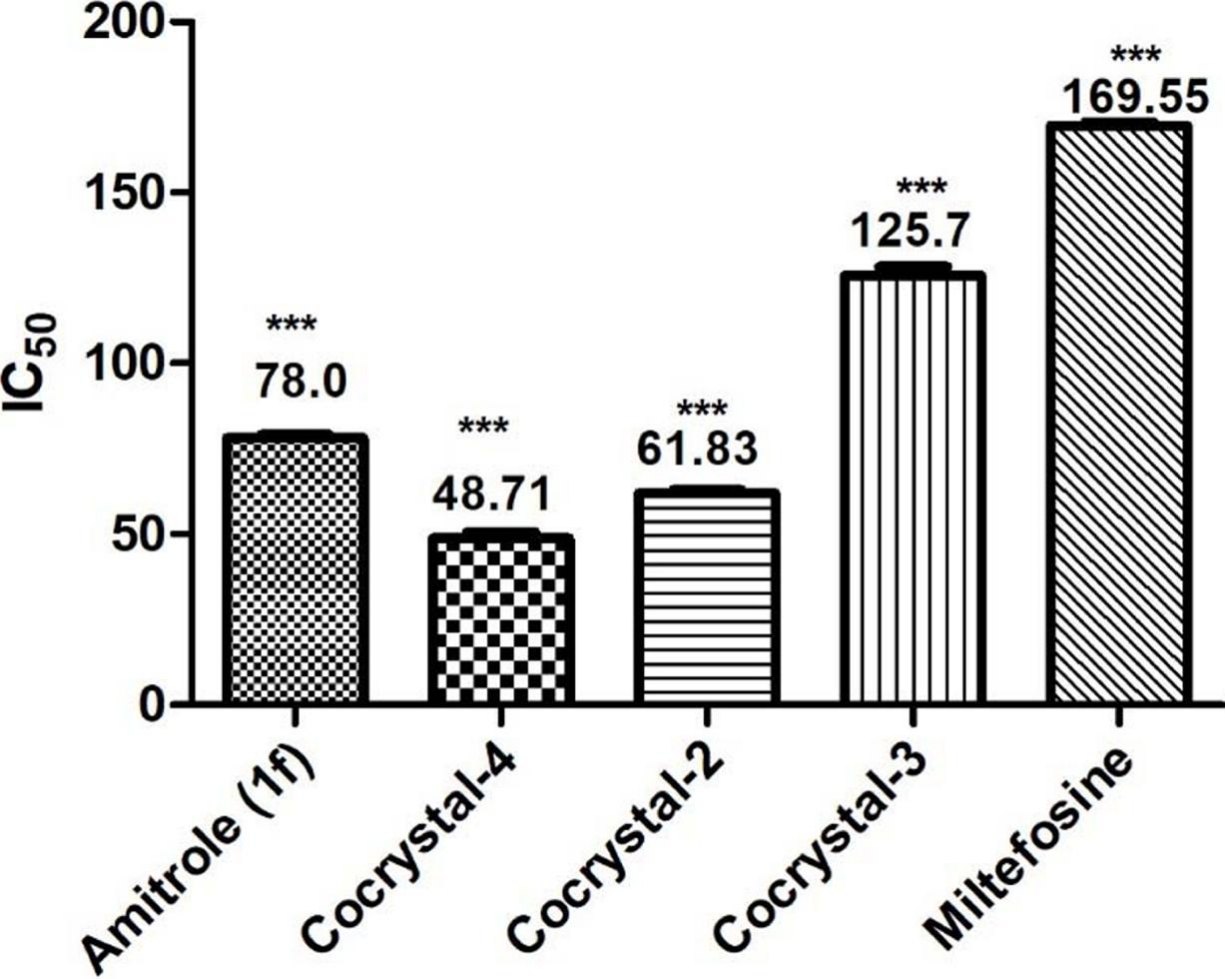
Comparative biological activities of **1f**, cocrystals **2**–**4** and MIL.

*In vitro* cytotoxicity of the synthesized cocrystals **CU**:**2**–**6** and their coformers **1b**–**1f** were evaluated in comparison with standard cyclo­heximide (IC_50_ = 0.8 ± 0.1 µ*M*) through MTT assay against 3T3 (normal mouse fibroblast) cell line. The results revealed that cocrystals **2**–**6** were non-cytotoxic (Table 3 ▸, Fig. 10 ▸).

## 4. Conclusions

Five new non-cytotoxic cocrystals of coumarin-3-carb­oxy­lic acid with pharmaceutically acceptable coformers were successfully synthesized via a neat grinding approach in a 1:1 stoichiometric ratio. Hirshfeld surface analysis demonstrated the impact of various non-covalent interactions towards the stability of the cocrystal in the solid state. Importantly, the anti-leishmanial activity evaluation against the MIL-resistant *L. tropica* revealed that synthesized cocrystals are more effective and non-toxic anti-leishmanial candidates compared with tested standard miltefosine against the resistant lines of clinical isolates of cutaneous leishmaniasis. Evidence that modification of supramolecular features via co-crystallization contributed towards anti-leishmanial activity is further supported by the fact that the physical mixtures (1:1) of API and amitrole were found to be inactive. Although further studies are required, the current work emphasizes the importance of cocrystallization of commercially available candidates with suitable coformers to enhance their therapeutic potential.

## Supplementary Material

Crystal structure: contains datablock(s) cocrystal_2, cocrystal_3, cocrystal_4, cocrystal_5, cocrystal_6. DOI: 10.1107/S2052252524001416/lq5052sup1.cif

Biological activity evaluation protocols and Figs. S1 to S14. DOI: 10.1107/S2052252524001416/lq5052sup2.pdf

CCDC references: 2156927, 2156930, 2156931, 2156932, 2233348

## Figures and Tables

**Table 1:** Crystallographic data and structure refinement parameters for cocrystals **2**–**6**

Cocrystal	**2**	**3**	**4**	**5**	**6**
Chemical formula	C_15_H_11_Br N_2_O_4_	C_15_H_10_F_3_ N_2_O_4_	C_12_H_9_N_4_O_4_	C_17_H_13_NO_6_	C_12_H_9_N_4_O_4_
API	**CU** (**1a**)	**CU** (**1a**)	**CU** (**1a**)	**CU** (**1a**)	**CU** (**1a**)
Coformer	2-Amino-3-bromo­pyridine (**1b**)	2-Amino-5-(tri­fluoro­methyl)­pyridine (**1c**)	2-Amino-6-methyl­pyridine (**1d**)	*p*-Amino­benzoic acid (**1e**)	Amitrole (**1f**)
*M*r	363.17	352.27	298.29	327.28	273.23
Temperature (K)	293 (2)	293 (2)	293 (2)	298 (2)	298 (2)
Wavelength (Å)	1.54178	1.54178	1.54178	1.54178	1.54178
Crystal system	Triclinic	Monoclinic	Monoclinic	Monoclinic	Orthorhombic
Space group	*P* 1	*C*2/*c*	*P*2_1_/*c*	*P*2_1_/*n*	*Pna*2
Unit-cell dimensions					
*a* (Å)	5.1722 (4)	28.3874 (10)	8.5818 (3)	11.6158 (3)	26.172 (3)
*b* (Å)	11.4102 (9)	4.9666 (2)	21.8411 (7)	7.6829 (2)	3.9741 (4)
*c* (Å)	12.6516 (11)	21.3260 (7)	7.7116 (2)	16.7602 (4)	11.4267 (11)
α (°)	76.401 (4)	90	90	90	90
β (°)	85.975 (4)	95.807 (2)	95.2730 (10)	103.2790	90
γ (°)	80.371 (4)	90	90	90	90
Volume (Å^3^)	715.12 (10)	2991.30 (19)	1439.31 (8)	1455.74 (6)	1188.5 (2)
*Z*	2	8	4	4	4
Density (calc.) (Mg m^−3^)	1.687	1.564	1.377	1.493	1.527
Absorption coefficient (mm^−1^)	4.116	1.204	0.836	0.971	1.007
*F*(000)	364	1440	624	680	564
Crystal size (mm)	0.09 × 0.12 × 0.22	0.09 × 0.11 × 0.16	0.04 × 0.09 × 0.23	0.14 × 0.15 × 0.37	0.03 × 0.05 × 0.15
Theta range (°)	3.596–68.233	3.129–68.243	4.048–68.284	6.368–68.176	3.377–68.230
Index ranges	−6 ≤ *h* ≤ 6	−34 ≤ *h* ≤ 34	−10 ≤ *h* ≤ 10	−13 ≤ *h* ≤ 13	−28 ≤ *h* ≤ 31
	−13 ≤ *k* ≤ 13	−5 ≤ *k* ≤ 5	−26 ≤ *k* ≤ 26	−9 ≤ *k* ≤ 9	−4 ≤ *k* ≤ 4
	−15 ≤ *l* ≤ 15	−25 ≤ *l* ≤ 25	−9 ≤ *l* ≤ 9	−20 ≤ *l* ≤ 20	−13 ≤ *l* ≤ 10
Reflections collected	19435	40009	38838	19255	4874
Independent reflections	2599 (*R*_int_ = 0.0543)	2718 (*R*_int_ = 0.0337)	2643 (*R*_int_ = 0.0700)	2642 (*R*_int_ = 0.0276)	1763 (*R*_int_ = 0.0948)
Completeness (%)	99.7	99.5	99.9	99.2	96.0
Refinement method	Full-matrix least-squares on *F* ^2^	Full-matrix least-squares on *F* ^2^	Full-matrix least-squares on *F* ^2^	Full-matrix least-squares on *F* ^2^	Full-matrix least-squares on *F* ^2^
Data/restraints/parameters	2599/0/201	2718/0/231	2643/0/209	2642/0/219	1763/1/194
Goodness-of-fit on *F* ^2^	1.086	1.037	1.089	1.057	1.063
Final *R* indices [*I* > 2σ (*I*)]	*R*1 = 0.0372, w*R*2 = 0.0916	*R*1 = 0.0315, w*R*2 = 0.0930	*R*1 = 0.0467, w*R*2 = 0.1196	*R*1 = 0.0385, w*R*2 = 0.1073	*R*1 = 0.0710, w*R*2 = 0.1514
*R* indices (all data)	*R*1 = 0.0450, w*R*2 = 0.0976	*R*1 = 0.0327, w*R*2 = 0.0942	*R*1 = 0.0586, w*R*2 = 0.1277	*R*1 = 0.0411, w*R*2 = 0.1099	*R*1 = 0.1295, w*R*2 = 0.1761
Extinction coefficient	0.0043 (6)	0.0022 (2)	0.0073 (8)	NA	0.4 (9)
Largest difference peak, hole (e Å^−3^)	0.435, −0.406	0.684, −0.650	0.411, −0.239	0.282, −0.231	NA
CCDC No.	2156930	2156932	2156931	2233348	2156927

**Table 2:** Hydrogen-bonding parameters in cocrystals **2**–**6**

Cocrystal	*D*—H⋯*A*	*D*—H (Å)	H⋯*A* (Å)	*D*⋯*A* (Å)	*D*—H⋯*A* (°)	Symmetry codes
**2**	C13—H13⋯O2^i^	0.93	2.50	3.097 (4)	122.5	(i) −*x*, −*y* + 1, −*z* + 1
	C14—H14⋯O)^i^	0.93	2.55	3.118 (4)	120.2	
	C15—H15⋯O4^ii^	0.93	2.36	3.232 (4)	156.0	(ii) −*x* + 1, −*y* + 1, −*z* + 1
	N2—H2B⋯O3^iii^	0.86	2.24	2.910 (3)	135.2	(iii) −*x*, −*y* + 2, −*z* + 1
	N2—H2A⋯O3^iv^	0.86	2.04	2.895 (4)	173.8	(iv) *x* + 2, *y*, *z* + 1
	O4—H4⋯N1^v^	0.82	1.78	2.577 (4)	164.8	(v) *x* − 2, *y*, *z* − 1
**3**	N2—H2B⋯O2^i^	0.86	2.16	2.9669 (16)	155.6	(i) *x* + 1/2, *y* − 1/2, *z*
	N2—H2B⋯O3^i^	0.86	2.28	2.8755 (14)	126.6	
	C12—H12⋯O2^i^	0.93	2.46	3.2087(16)	138.1	
	C8—H8⋯O1^ii^	0.93	2.60	3.5228 (16)	170.4	(ii) −*x* + 1/2, *y* + 1/2, −*z* + 3/2
	N2—H2A⋯O3^iii^	0.86	2.08	2.9292 (15)	168.4	(iii) −*x* + 1, −*y*, −*z* + 1
**4**	C16—H16B⋯O4^i^	0.96	2.62	3.533 (3)	158.8	(i) −*x* + 1, *y* + 1/2, −*z* + 1/2
	O3—H3A⋯N1^ii^	0.82	1.86	2.658 (2)	165.1	(ii) −*x* + 1, *y* − 1/2, −*z* + 3/2
	C12—H12⋯O2^iii^	0.93	2.62	3.425 (3)	144.8	(iii) *x* + 1, −*y* + 1/2, *z* + 1/2
	C16—H16A⋯O3^iv^	0.96	2.60	3.410 (3)	141.8	(iv) −*x* + 1, *y* + 1/2, −*z* + 3/2
	N2—H2A⋯O4^iii^	0.89 (3)	2.01 (3)	2.817 (3)	150 (2)	(iii) *x* + 1, −*y* + 1/2, *z* + 1/2
**5**	O4—H4A⋯O6^i^	0.82	1.85	2.6581 (14)	167.5	(i) −*x*, −*y*, −*z* + 2
	O5—H5A⋯O3^ii^	0.82	1.84	2.6417 (14)	166.9	
	N1—H1B⋯O6^ii^	0.86	2.58	3.4143 (17)	163.4	(ii) −*x* + 1/2, *y* + 1/2, −*z* + 5/2
	C16—H16⋯O1^iii^	0.93	2.55	3.4789 (16)	174.5	(iii) −*x* + 1, −*y* + 1, −*z* + 2
	N1—H1A⋯O2^iii^	0.86	2.32	3.1762 (16)	176.0	
**6**	O4—H4⋯N2^i^	0.82	1.90	2.675 (9)	157.0	(i) −*x* + 1/2, *y* − 1/2, *z* − 1/2
	C8—H8⋯O3^ii^	0.93	2.59	3.334 (12)	137.7	(ii) −*x* + 1, −*y*, *z* + 1/2
	N4—H4A⋯O3^iii^	1.04 (11)	1.80 (11)	2.799 (11)	160 (8)	(iii) *x* + 1/2, *y* + 1/2, *z* + 1/2
	N4—H4B⋯N1^iv^	0.87 (11)	2.23 (11)	3.067 (9)	163 (9)	(iv) −*x* + 1/2, *y* + 1/2, *z* − 1/2
	N3—H3A⋯O2	0.72 (11)	2.51 (11)	2.957 (11)	121 (10)	
	N3—H3A⋯O4	0.72 (11)	2.00 (12)	2.686 (11)	158 (11)	

**Table:** 

	Anti-leishmanial activity against MIL-resistant *L. tropica* via MTT assay		
Sample	(IC_50_ = µ*M* ± SEM)	*p*-value	Cytotoxic activity 3T3 cell line (IC_50_ = µ*M* ± SEM)
Coumarin-3-carboxilic acid (**CU**) (**1a**)	NA	NA	Non-cytotoxic
2-Amino-3-bromo­pyridine (**1b**)	NA	NA	Non-cytotoxic
2-Amino-5-(tri­fluoro­methyl)­pyridine (**1c**)	NA	NA	Non-cytotoxic
2-Amino-6-methyl­pyridine (**1d**)	NA	NA	Non-cytotoxic
*p*-Amino­benzoic acid (**1e**)	NA	NA	Non-cytotoxic
Amitrole (**1f**)	78.0 ± 0.96	<0.05	Non-cytotoxic
Cocrystal **2**	61.83 ± 0.59	<0.05	Non-cytotoxic
Cocrystal **3**	125.7 ± 1.15	<0.05	Non-cytotoxic
Cocrystal **4**	48.71 ± 0.75	<0.05	Non-cytotoxic
Cocrystal **5**	NA	<0.05	Non-cytotoxic
Cocrystal **6**	NA	NA	Non-cytotoxic
Mixture of **1a**:**1b** (1:1)	NA	NA	Not evaluated
Mixture of **1a**:**1c** (1:1)	NA	NA	Not evaluated
Mixture of **1a**:**1d** (1:1)	NA	NA	Not evaluated
Mixture of **1a**:**1e** (1:1)	NA	NA	Not evaluated
Mixture of **1a**:**1f** (1:1)	NA	NA	Not evaluated
Standards	169.55 ± 0.078 miltefosine	<0.05	0.8 ± 0.14 cyclo­hexamide

**Table:** 

Tukey’s Multiple Comparison Test (Fig. 10 ▸)	Significant? *P* < 0.05	Summary
Amitrole (**1f**) versus MIL	Yes	***
Cocrystal **2** versus MIL	Yes	***
Cocrystal **3** versus MIL	Yes	***
Cocrystal **4** versus MIL	Yes	***
