# Supplementary material for: Cocrystals of a coumarin derivative: an efficient approach towards anti-leishmanial cocrystals against MIL-resistant Leishmania tropica
Source: IUCrJ. 2024 Mar 1;11(Pt 2):224–36. doi: 10.1107/S2052252524001416 (PMC10916291; doi:10.1107/S2052252524001416)
Supplement: Supplementary file 2 [file m-11-00224-sup2.pdf]

IUCrJ

Volume 11 (2024)

Supporting information for article:

Cocrystals of coumarin derivative: an efficient approach towards anti-leishmanial cocrystals against MIL-resistant *Leishmania tropica*

Muhammad Shahbaz, Saba Farooq, M. Iqbal Choudhary and Sammer Yousuf

Supporting Information

Biological activities evaluations

1. **MIL-resistant *L. tropica* assay protocol**

Materials and Methods:

Chemicals:

Miltefosine (hexadecylphosphocholine) (LEAP Chem), RPMI-1640 culture medium (Gibco), supplemented with 10% (v/v) heat-inactivated fetal bovine serum (FBS) (Gibco), 2 mM L-glutamine, 1% penicillin/ streptomycin (Sigma), DMSO (ALC labscan), and Trypan blue (Aldrich).

Clinical Isolate and Generation of MIL resistant line:

Clinical isolate (*L. tropica* promastigote) from the endemic area of Islamabad (Pakistan) was obtained from a patient. Promastigote cultures were maintained at 26 °C in RPMI medium, containing 15 % FBS and 1 % penicillin–streptomycin mixture. Clinical isolate was characterized by sequencing**.**

Generation of Miltefosine-unresponsive strain was carried under high MIL pressure by *in vitro* passage with stepwise increase in the MIL concentration. At each step, parasites were cultured, and passaged every 3–4 days at an initial concentration of $5x10^5$ promastigotes/mL in order to achieve the stable growth, as compared to the wild type isolate. Growth rates were measured for resistant populations and compared with the WT strain. Parasites were counted at an initial concentration of 5×10^5 parasites/mL, and growth was measured daily using a Neubauer chamber until the population reached stationary phase. Furthermore, the fluorescence microscopic investigations *via* DAPI stain were also carried out to supplement the study, and for confirmations.

Promastigote Growth Inhibition Assay

For the estimation of particular concertation at which tested compound caused 50% inhibition (IC_{50}) of resistant cell proliferation concerning untreated controls, the MTT assay was employed. The promastigotes in their log phase were used in 96-well plates. 1×10^6 wild-type and resistant

promastigotes were dispensed in 96-well plates, and incubated with tested compounds at a range concentration of 200–10 µM (stock concentration 1mg/mL and the working concentrations started from 250 µg/mL) at 27°C for 72 h. After incubation of 72 h, MTT dye (3-(4,5-dimethylthiazol-2-yl)-2,5-diphenyl-2H-tetrazolium bromide) was added and further incubate it for 3–4 h. Amphotericin B, pantamidine and miltefosine were used as the positive control, untreated promastigotes were used as negative control moreover DMSO control was also added in the study as a stock solution of the compounds that were prepared in DMSO. All the experiments were performed in triplicates. After completion of the experiment the absorbance by Multiskan ascent plate reader at 452 nm and percent inhibition was calculated by the following formula.

Cytotoxic concentration (%) = [100- (Absorbance of test)/ (Absorbance of solvent control) ×100] %.

Table S1: Thus IC_{50s} were determined as follows:

Coumarin-3-carboxylic acid: 2-amino-3-bromopyridine (**2**)		
**Concentrations**	**Percent Inhibitions**	**IC_{50}**
137.6	78.24	61.83 ± 0.59 µM
68.83	55.31	
34.41	29.17	
Coumarin-3-carboxylic acid:2-amino-6-methylpyridine (**3**)		
141.9	54.05	125.7 ± 1.15 µM
70.97	36.29	
35.48	20.72	
Coumarin-3-carboxylic acid:2-amino-6-methylpyridine (**4**)		
167.6	82.11	48.71 ± 0.75 µM
83.18	59.45	
41.9	48.13	
Amitrole (**1f**)		
187	63.01	78.0 ± 0.96 µM
93.88	53.1	
46.95	44.0	

2. Cytotoxicity assay Protocol:

Cytotoxicity of compounds was evaluated in 96-well flat-bottomed micro plates by using the standard MTT (3-[4, 5-dimethylthiazole-2-yl]-2, 5-diphenyl-tetrazolium bromide) colorimetric assay. For this purpose, 3T3 (mouse fibroblast) cells were cultured in Dulbecco's Modified Eagle Medium, supplemented with 5% of fetal bovine serum (FBS), 100 *IU/mL* of penicillin and 100 *µg/mL* of streptomycin in 75 cm^2 flasks, and kept in 5% CO_2 incubator at 37° C. Exponentially growing cells were harvested, counted with haemocytometer, and diluted with a particular medium. Cell culture with the concentration of $5x10^4$ *cells/mL* was prepared and introduced (100 *µL*/well) into 96-well plates. After overnight incubation, medium was removed and 200 *µL* of fresh medium was added with different concentrations of compounds (1-30 *µM*). After 48 hrs. 200 *µL* MTT (0.5 *mg/mL*) was added to each well, and incubated further for 4 hrs. Subsequently, 100 *µL* of DMSO was added to each well. The extent of MTT reduction to formazan within cells was calculated by measuring the absorbance at 540 nm, using a micro-plate reader (Spectra Max plus, Molecular Devices, USA). The cytotoxicity was recorded as a concentration causing 50% growth inhibition (IC_{50}) of 3T3 cells. The percent inhibition was calculated by using the following formula:

% Inhibition = 100-((mean of O.D of test compound – mean of O.D of negative control)/ (mean of O.D. of positive control – mean of O.D of negative control)*100).

The results (% inhibition) were processed by using Soft- Max Pro software (Molecular Devices, USA).

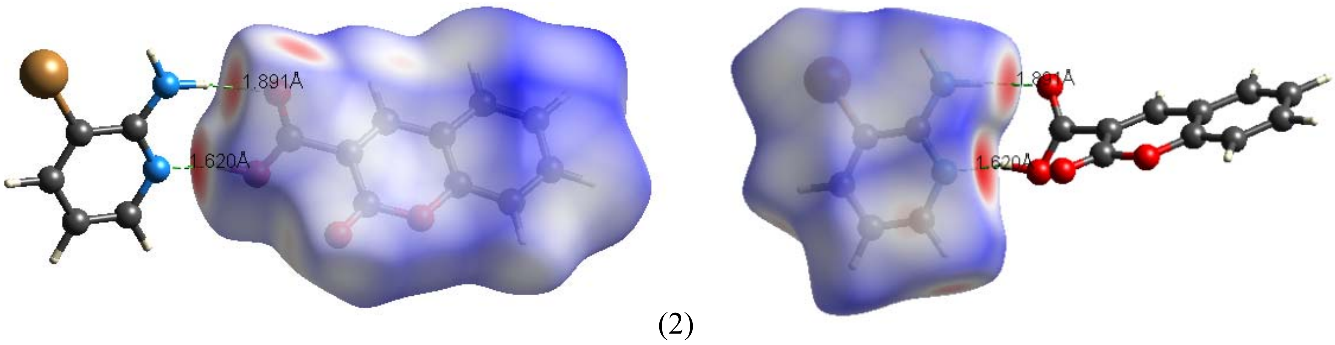

(2)

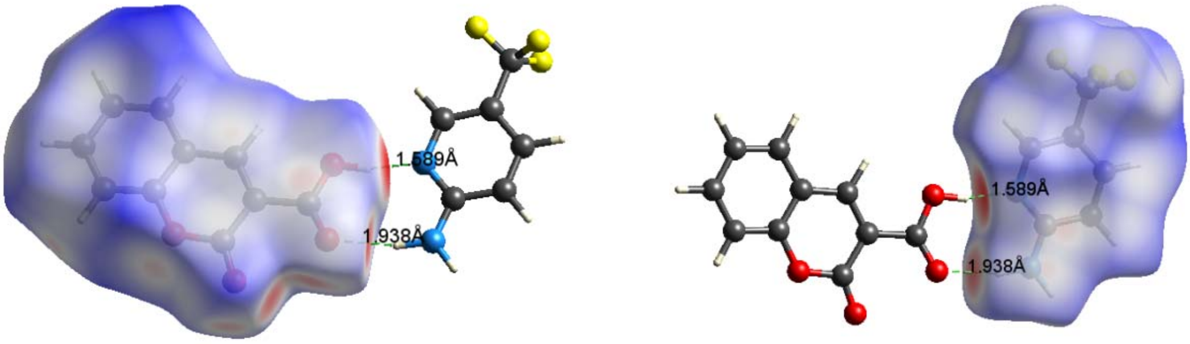

(3)

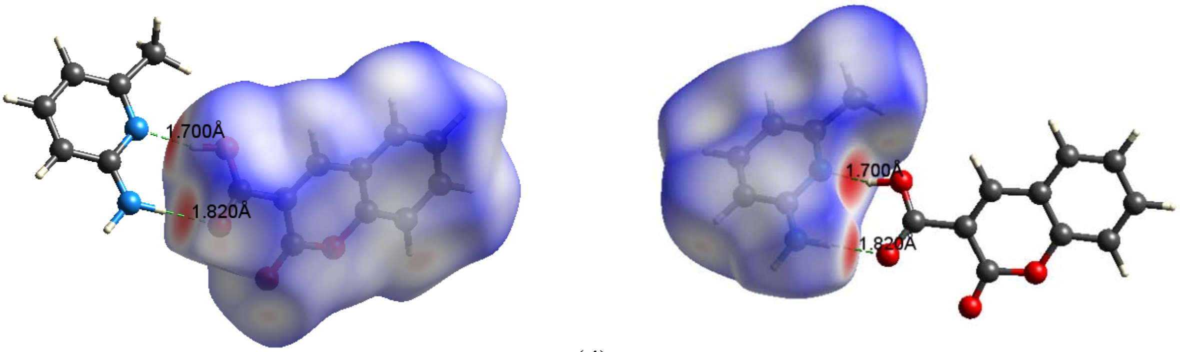

(4)

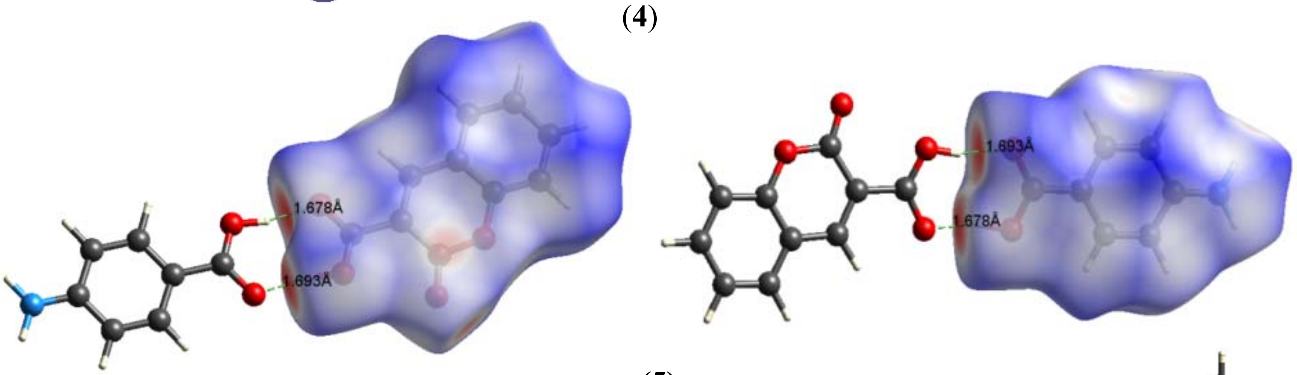

(5)

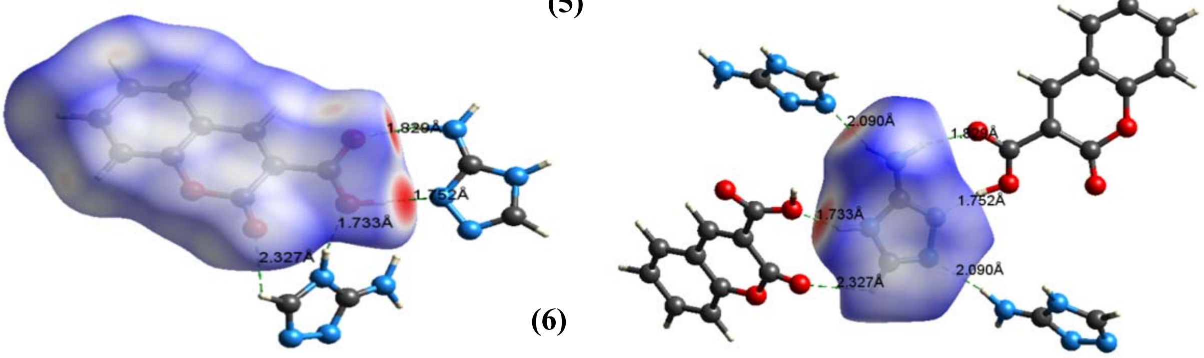

(6)

Figure 1S: Two-dimentional packing interactions generated over Hirshfeld surfaces for the syntheized cocrystals **2-6**.

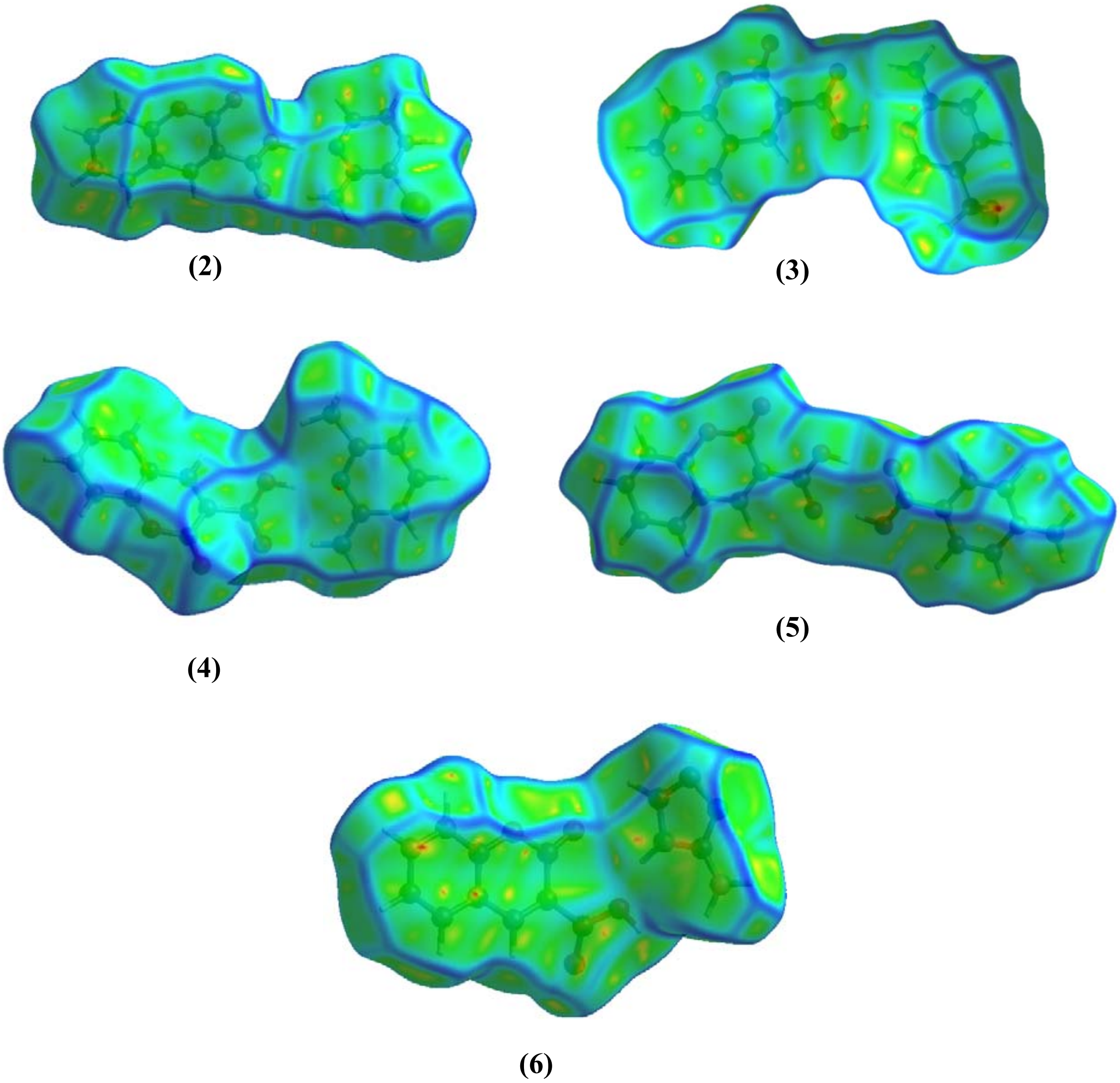

Figure 2S: Two-dimensional curvedness surface generated over Hirshfeld surface of synthesized cocrystals **2-6**.

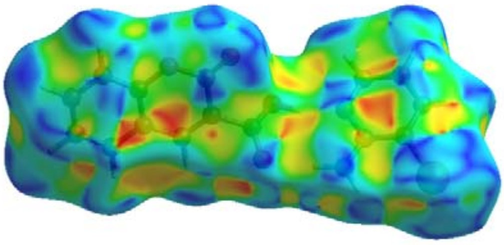

(2)

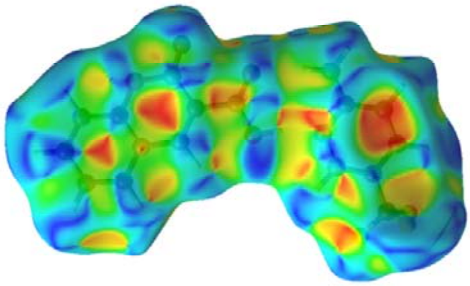

(3)

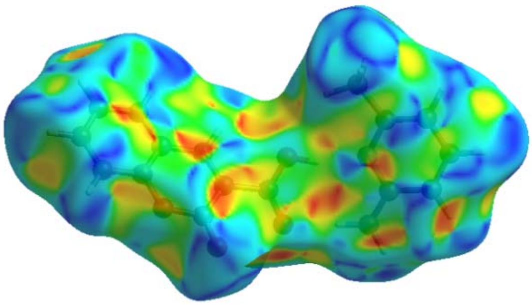

(4)

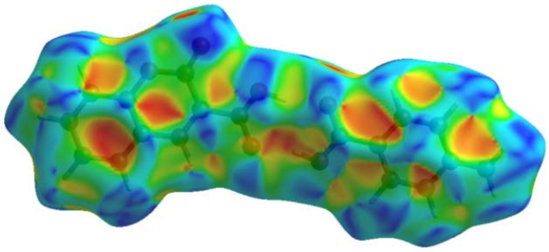

(5)

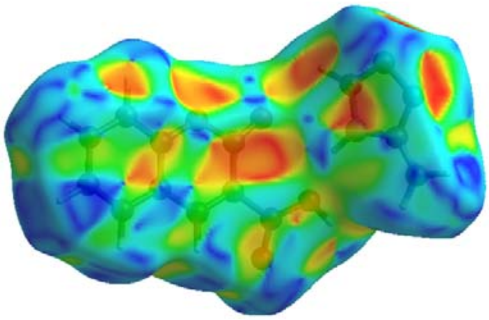

(6)

Figure 3S: Two-dimensional shape index surface generated over Hirshfeld surface of synthesized cocrystals **2-6**.

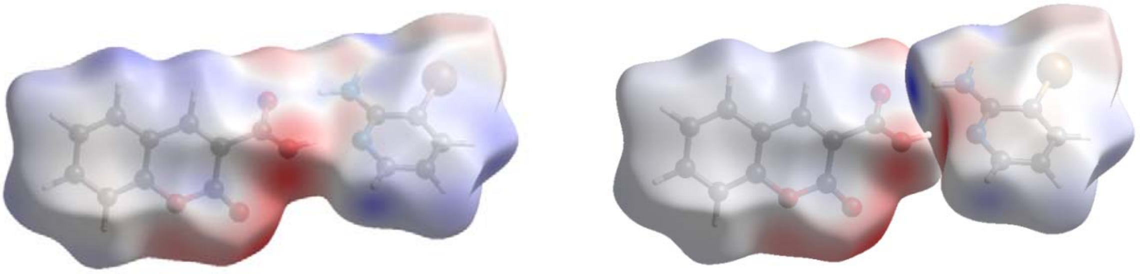

(**2**)

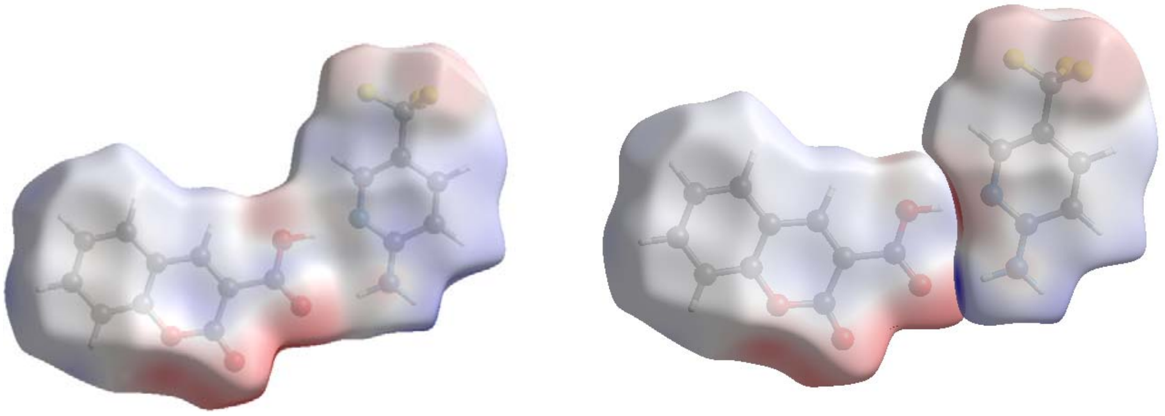

(**3**)

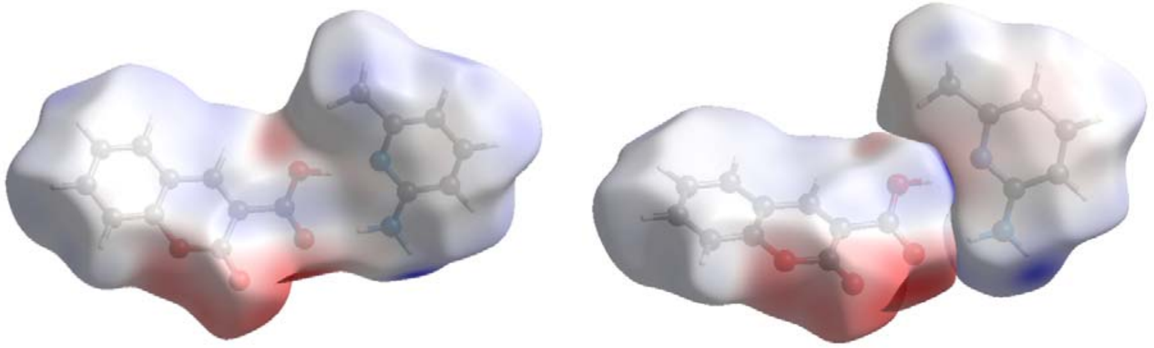

(**4**)

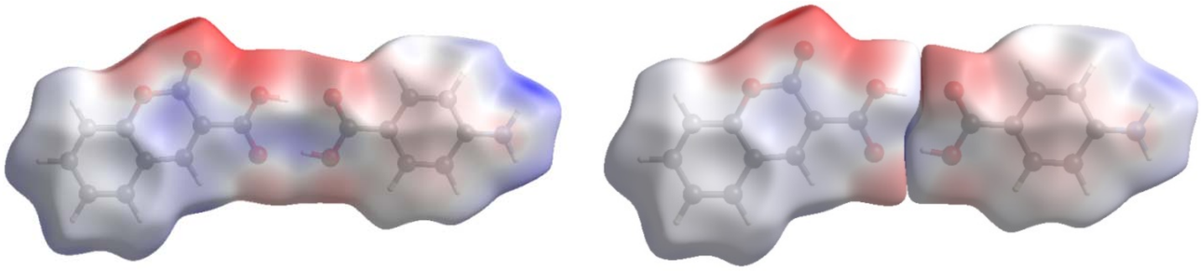

(**5**)

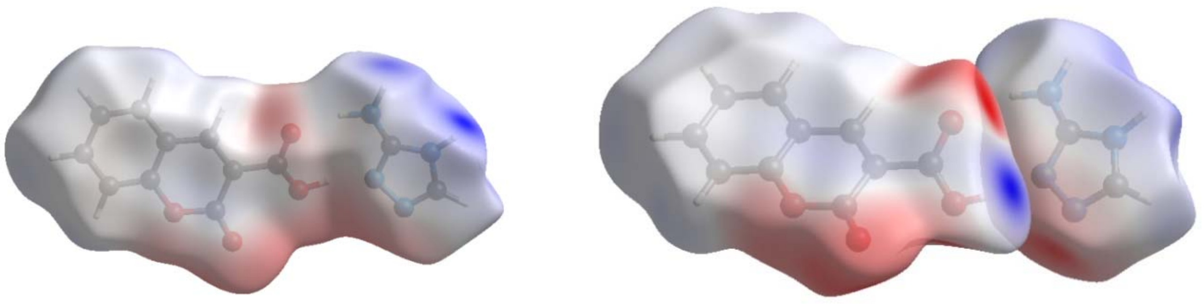

(6)

Figure 4S: Two-dimensional electrostatic potential (the red and blue regions represent negative and positive electrostatic potentials, respectively) mapped over Hirshfeld surface of synthesized cocrystals (**2-6**).

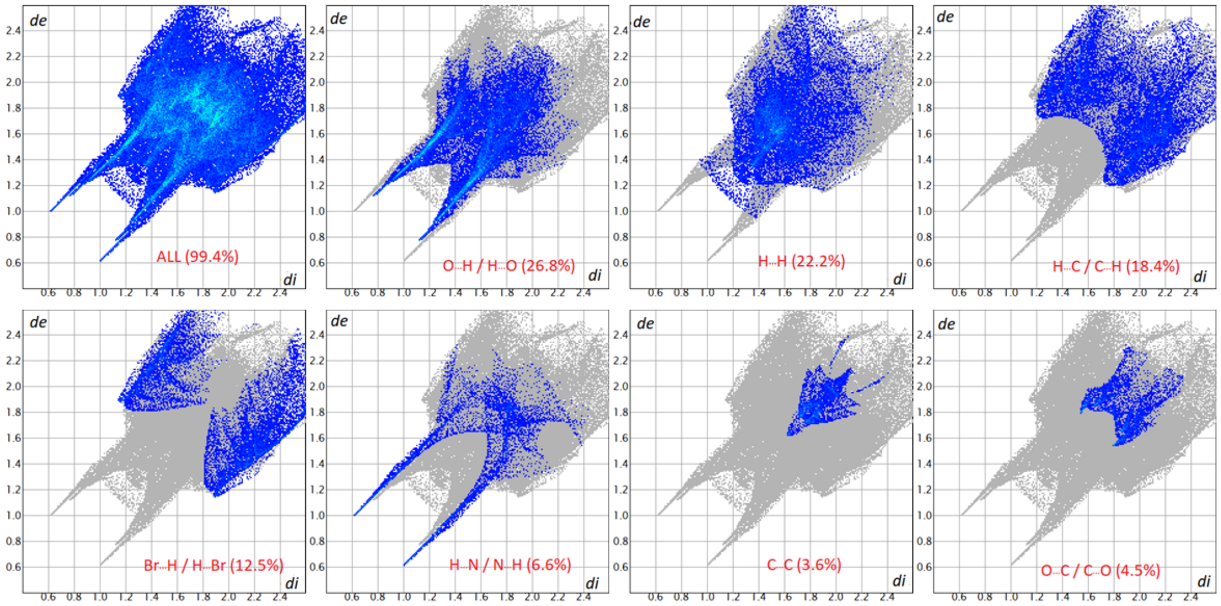

Figure 5S: Two dimensional finger print plots, percentage contributions of the contents of cocrystal **2**.

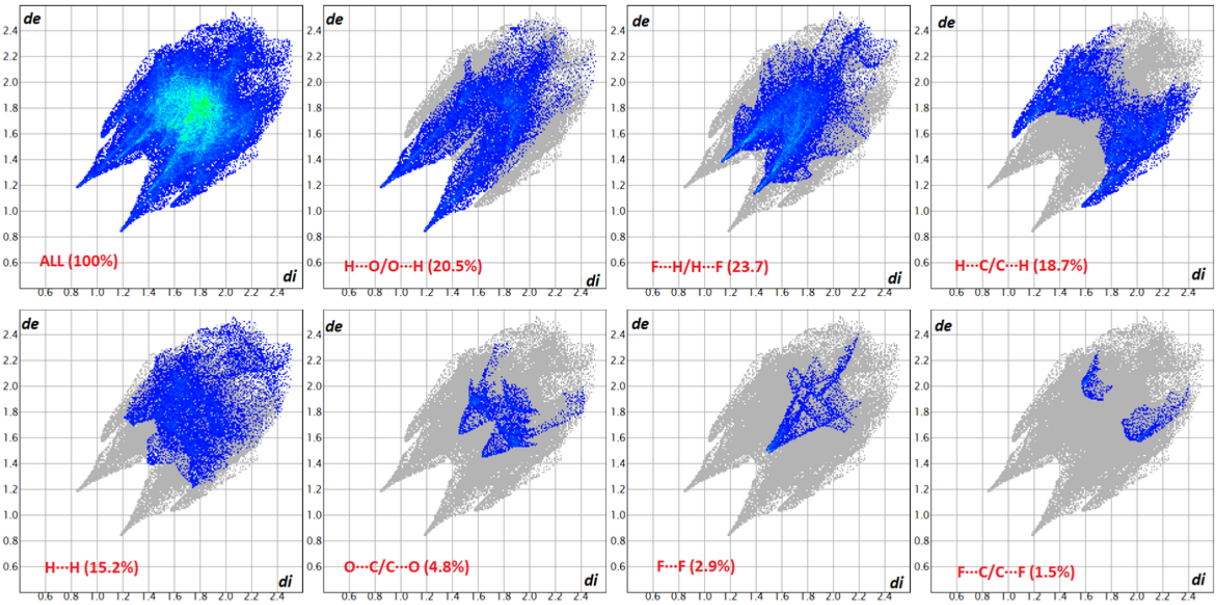

Figure 6S: Two dimensional finger print plots, percentage contributions of the contents of cocrystal **3**.

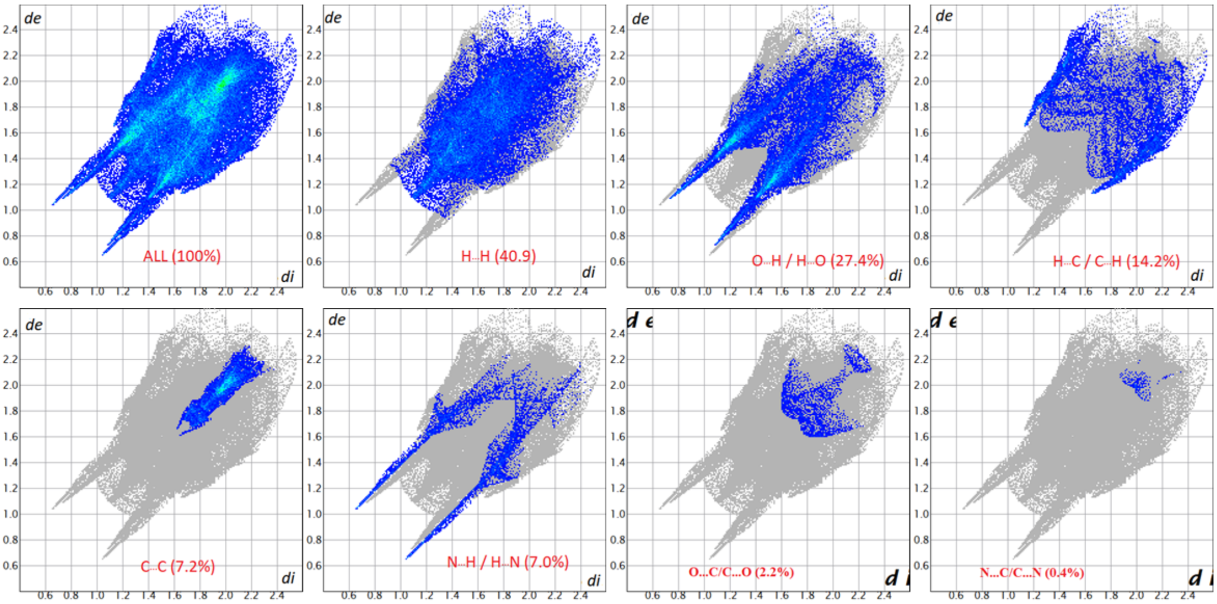

Figure 7S: Two dimensional finger print plots, percentage contributions of the contents of cocrystal **4**.

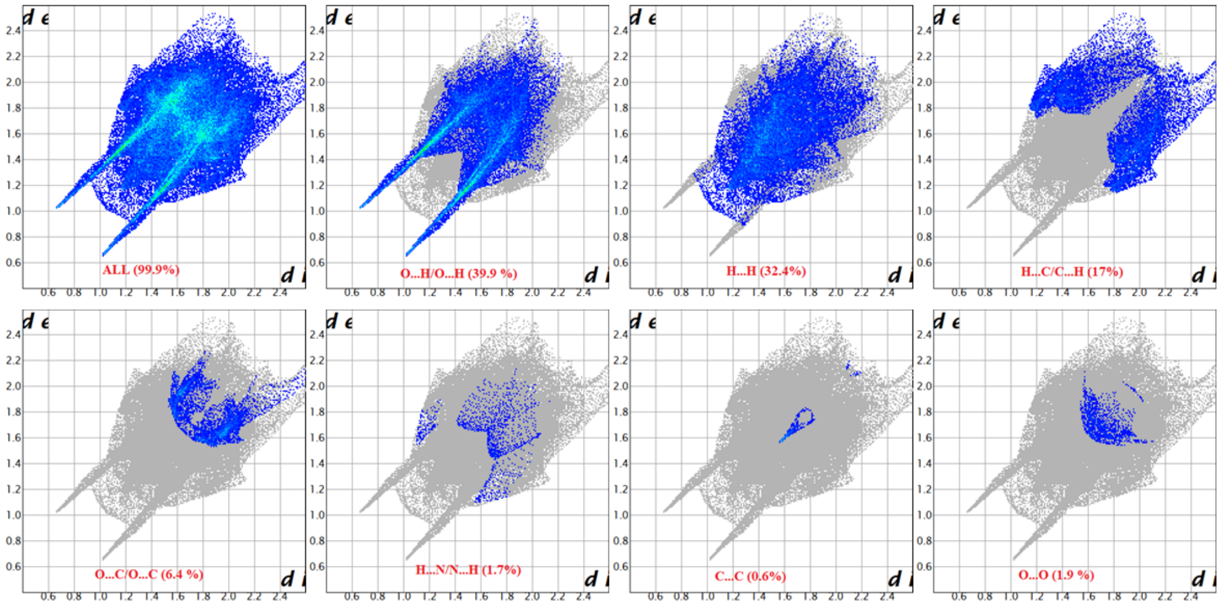

Figure 8S: Two dimensional finger print plots, percentage contributions of the contents of cocrystal **5**.

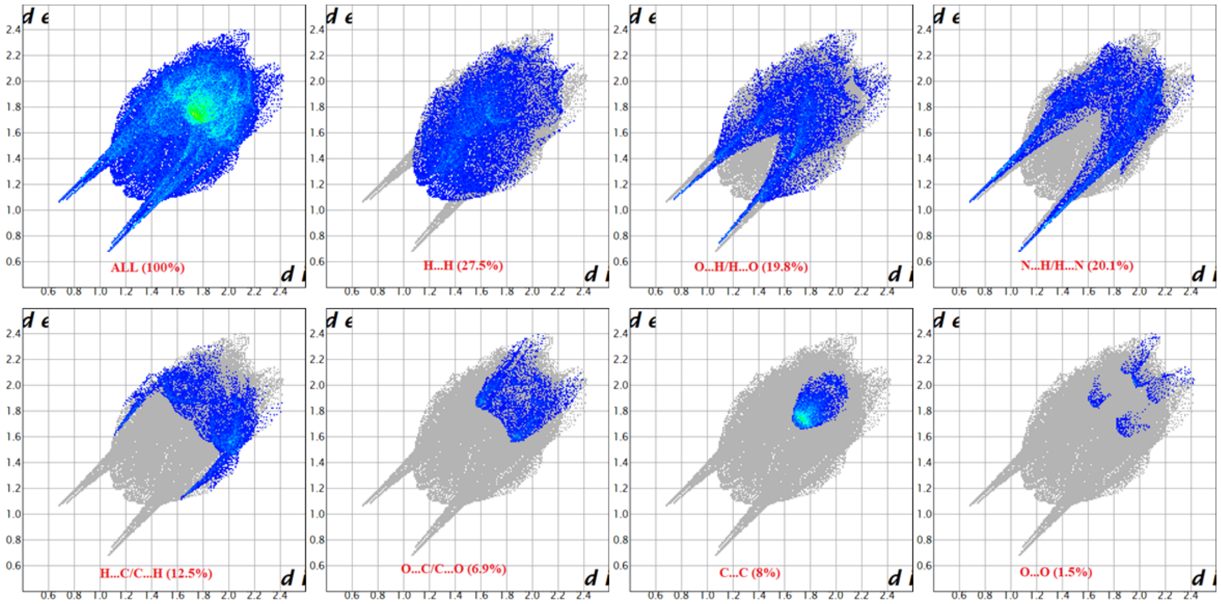

Figure 9S: Two dimensional finger print plots, percentage contributions of the contents of cocrystal **6**.

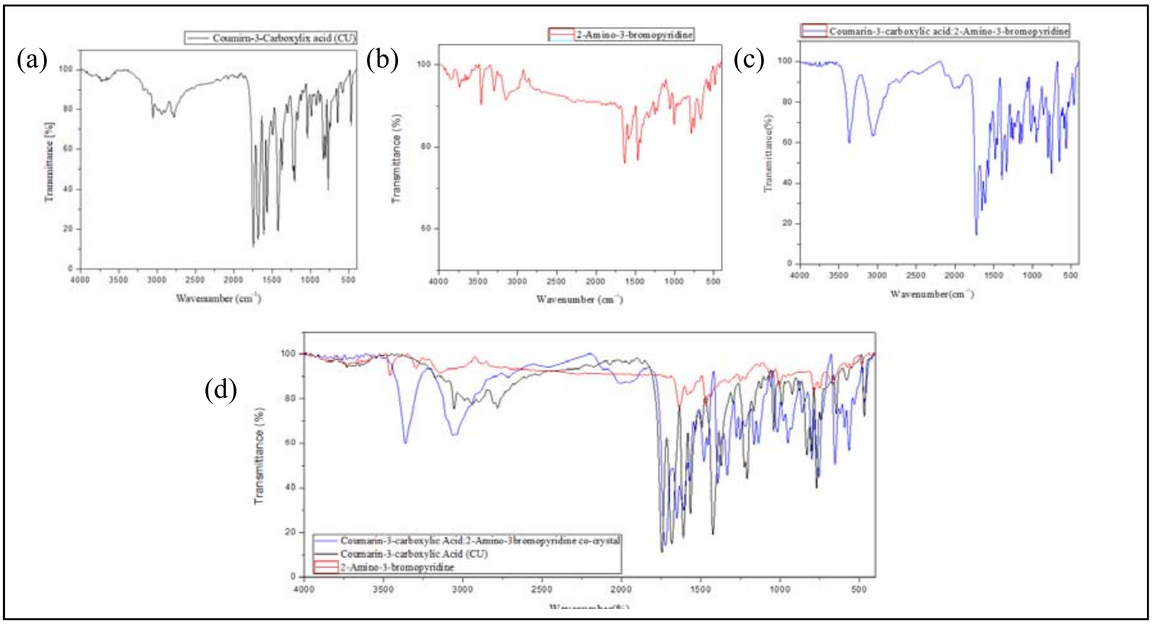

Figure 10S: FT-IR Spectra of (a) coumarin-3-carboxylic acid **CU** (**1a**), (**b**) 2-amino-3-bromopyridine (**1b**),(**c**) (CU:2-amino-3-bromopyridine) cocrystal **2**, and (**d**) their comparison.

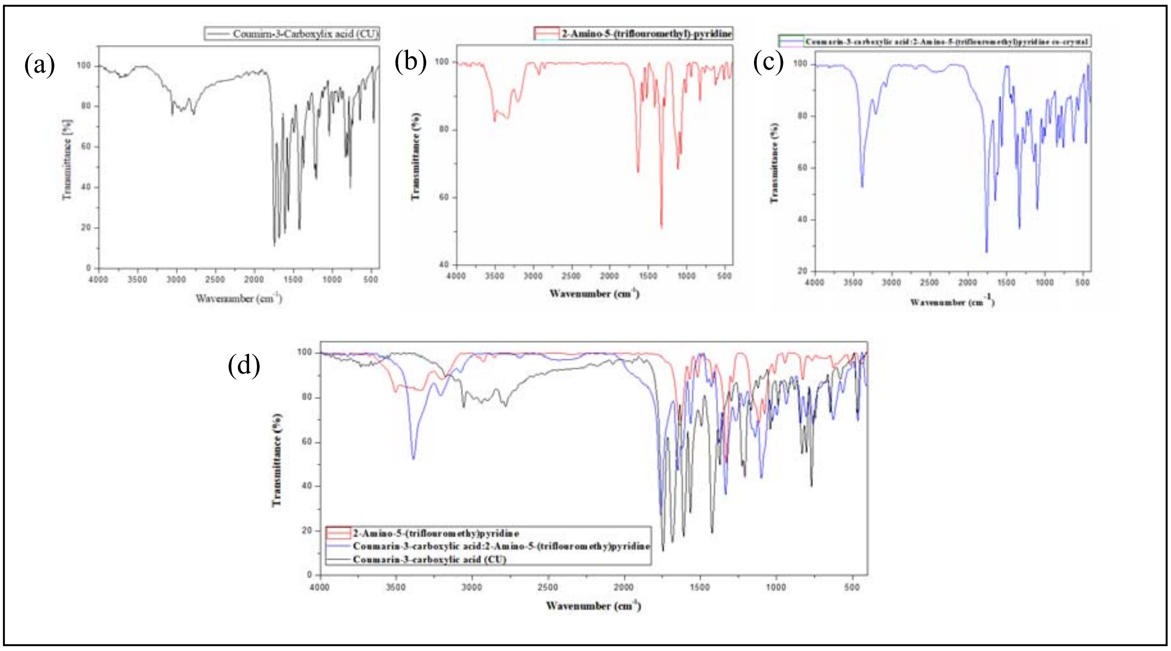

Figure 11S: FT-IR Spectra of (**a**) coumarin-3-carboxylic acid **CU** (**1a**), (**b**) 2-amino-5-(trifluoromethyl)pyridine (**1c**), (**c**) (CU:2-amino-5-(trifluoromethyl)pyridine) cocrystal **3**, and (**d**) their comparison.

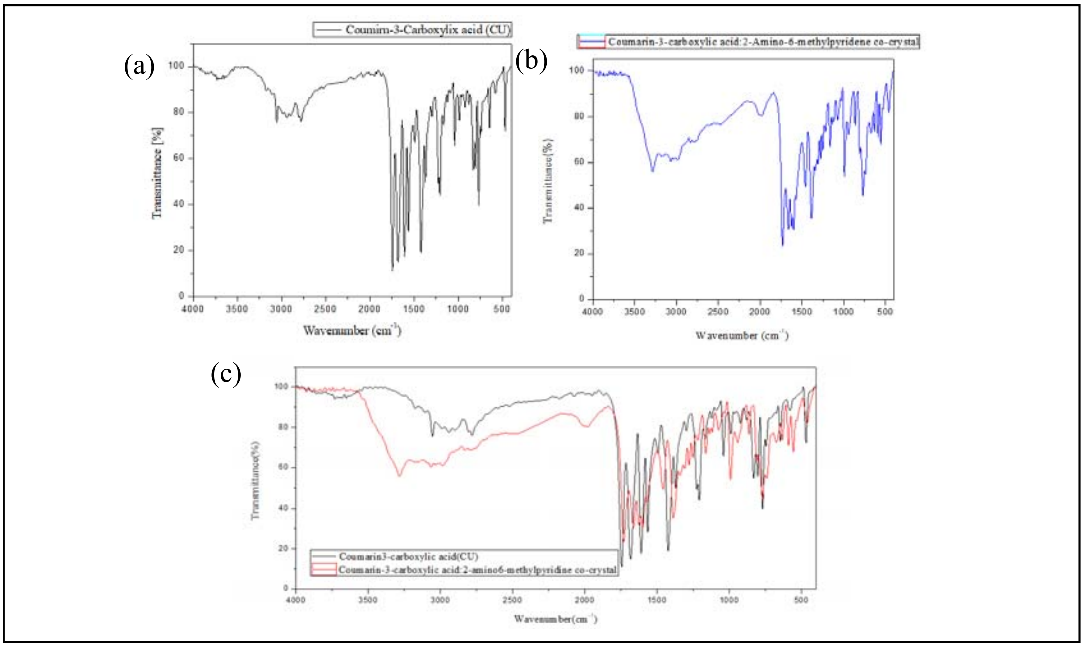

Figure 12S: FT-IR Spectra of (a) coumarin-3-carboxylic acid CU (**1a**), (b) (CU:2-amino-6-methylpyridine) cocrystal **4**, and (c) their comparison.

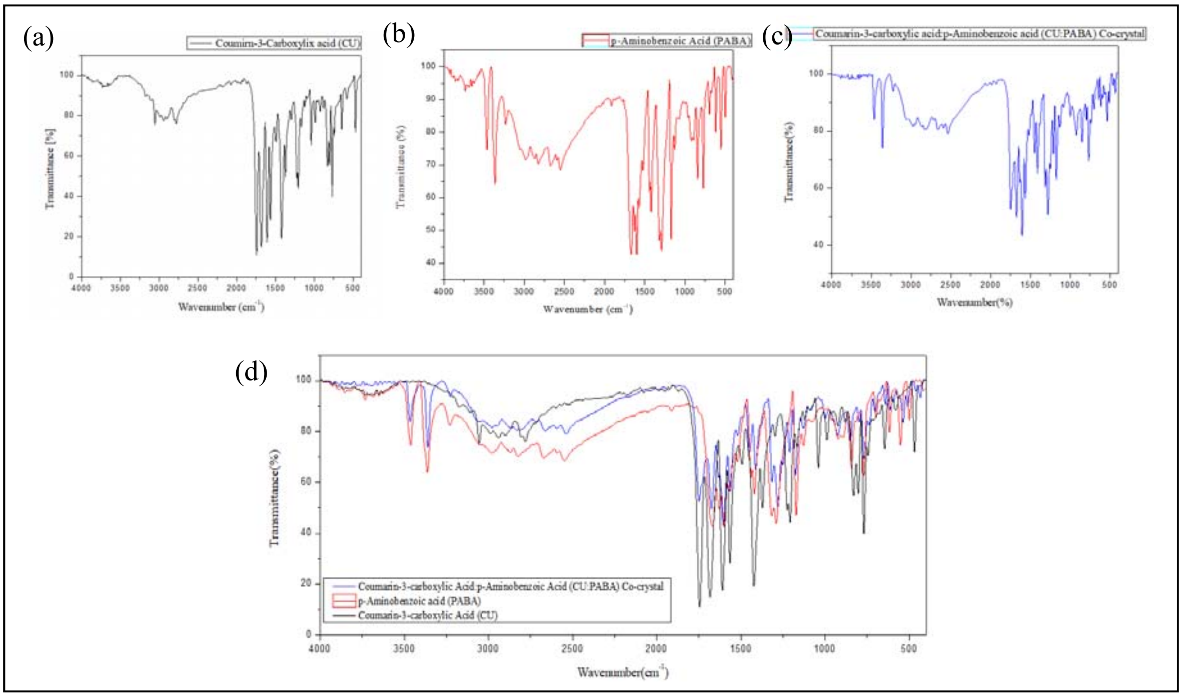

Figure 13S: FT-IR Spectra of (a) coumarin-3-carboxylic acid (**CU**) (**1a**), (b) *p*-aminobenzoic acid (**1d**) (c) (CU:PABA) cocrystal **5**, and (d) their comparison.

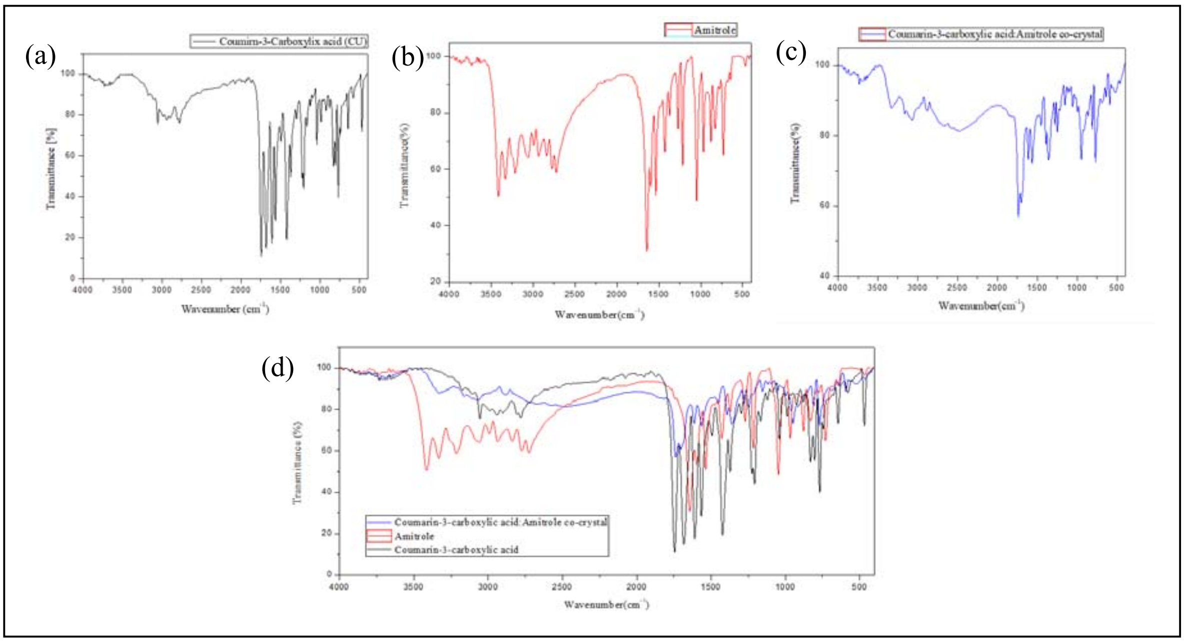

Figure 14S: FT-IR Spectra of (a) coumarin-3-carboxylic acid (**CU**), (**1a**) (b) amitrole (**1f**), (c) (CU:amitrole) cocrystal **6**, and (d) their comparison.

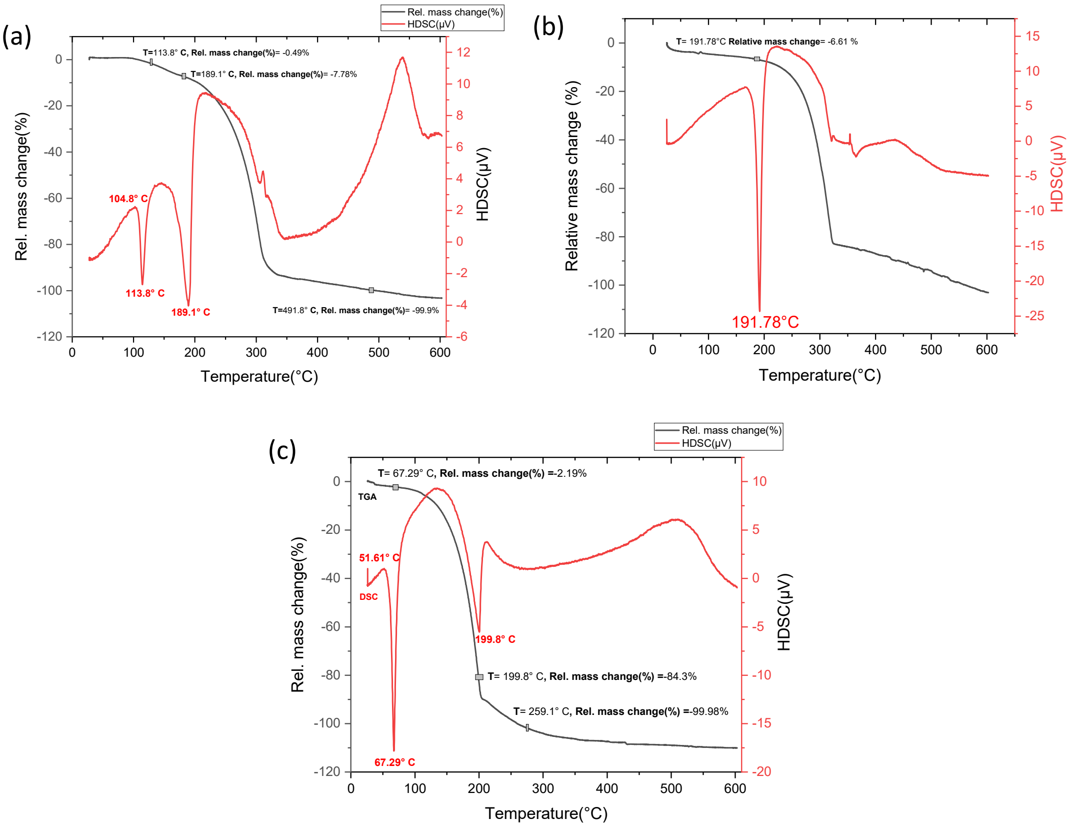

Figure 15S: DSC/TGA Spectra of (a) cocrystal **2**, (b) **CU** (**1a**) and, (C) 2-amino-3-bromopyridine (**1b**).

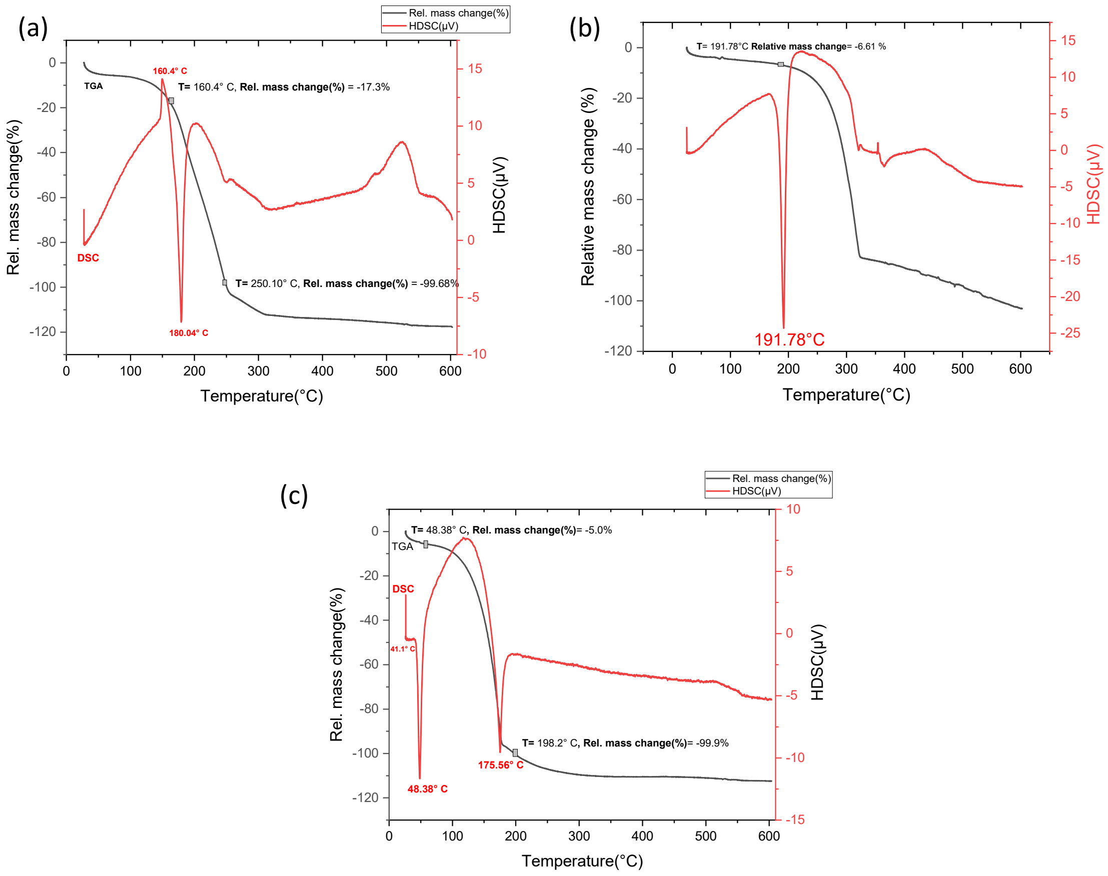

Figure 16S: DSC/TGA Spectra of (a) cocrystal **3**, (b) **CU** (**1a**), and (C) 2-amino-5-(trifluoromethyl)pyridine (**1c**).

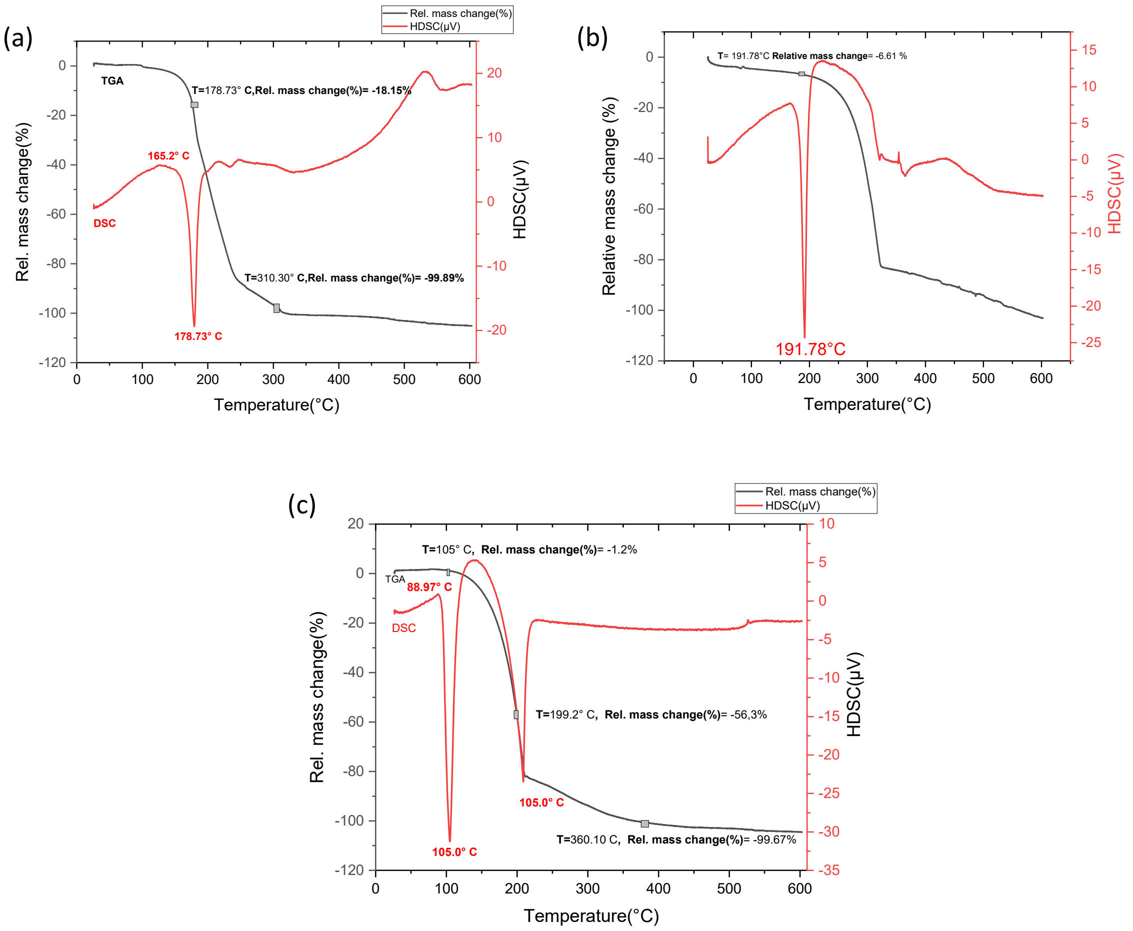

Figure 17S: DSC/TGA Spectra of (a) cocrystal **4**, (b) **CU** (**1a**), and (C) 2-amino-6-methylpyridine (**1d**).

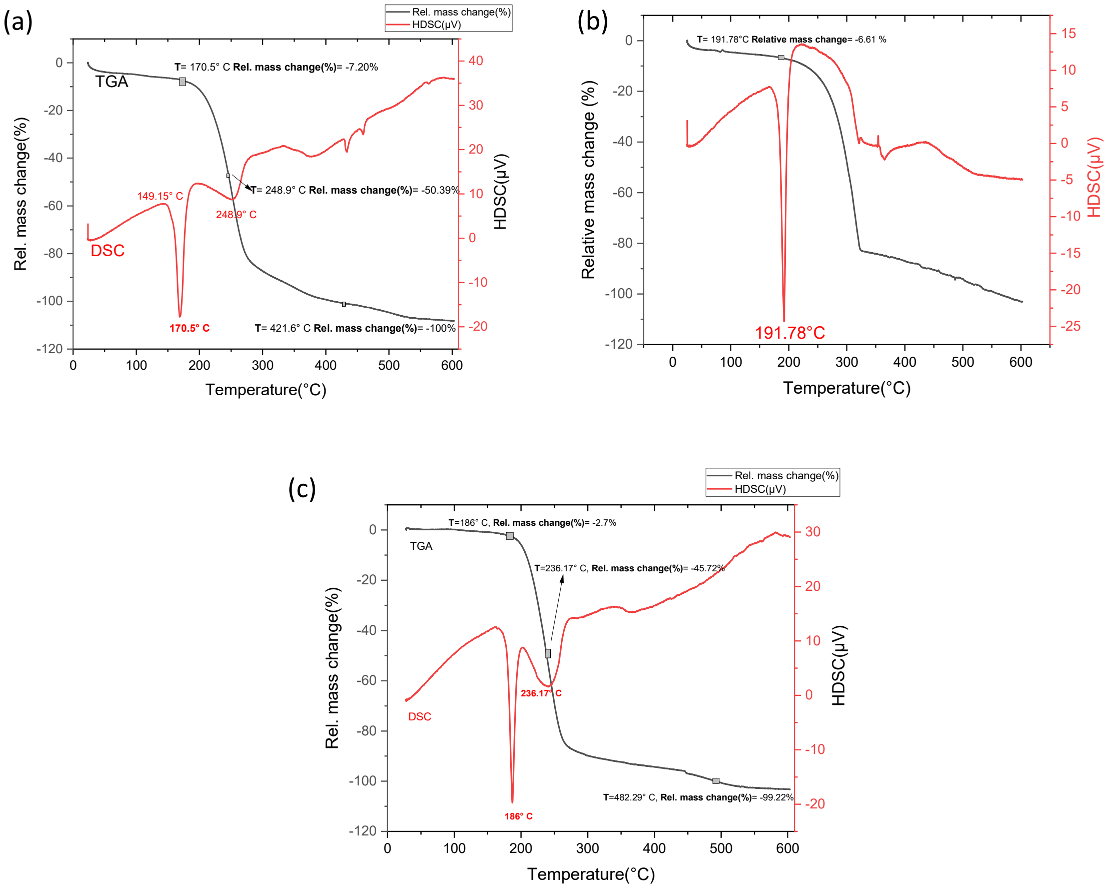

Figure 18S: DSC/TGA Spectra of (a) cocrystal **5**, (b) **CU** (**1a**), and (C) PABA (**1e**).

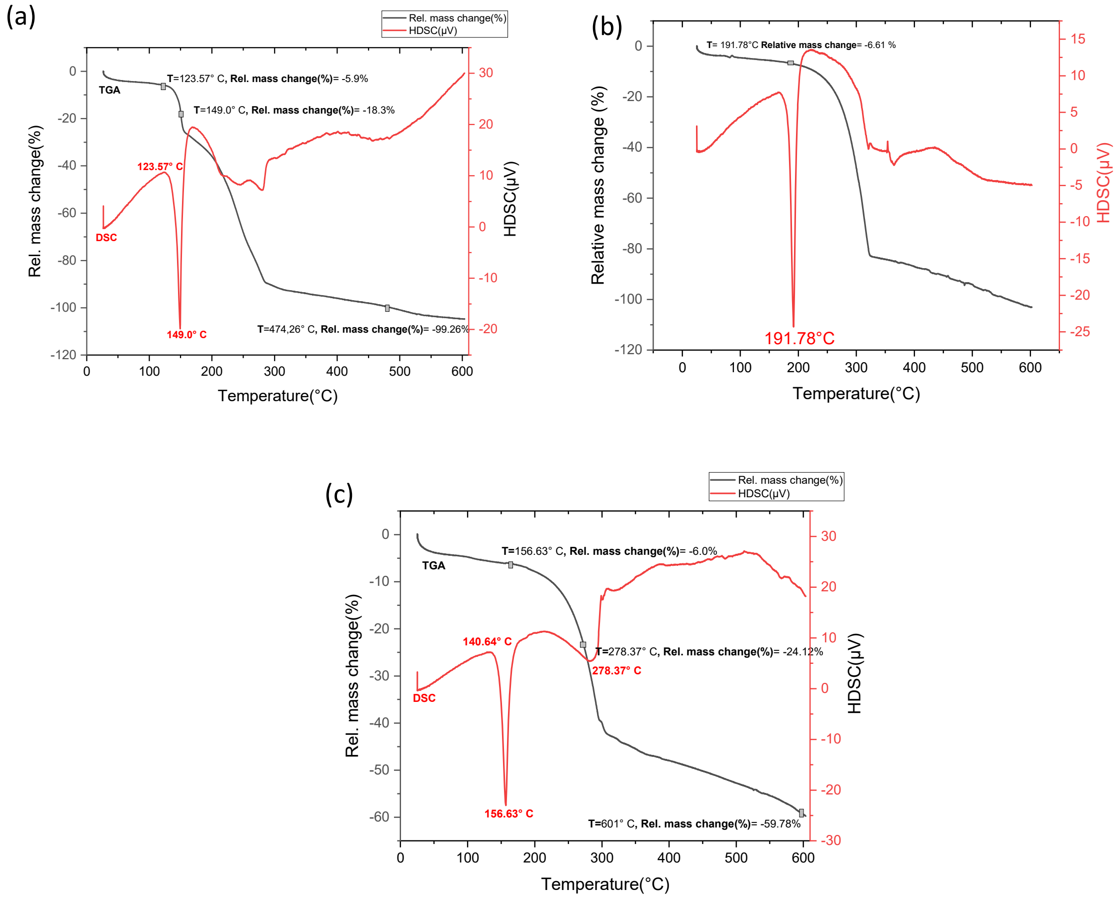

Figure 19S: DSC/TGA Spectra of (a) cocrystal **6**, (b) **CU** (**1a**), and (C) amitrole (**1f**).

S-2: List of coformers failed to be co-crystallizwd with CU 1

S. No	Name and structure of compound	S. No	Name and structure of compound
1	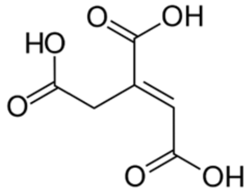 *trans*-aconitic acid	6	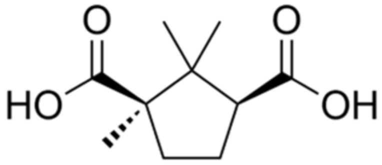 Camphoric acid
2	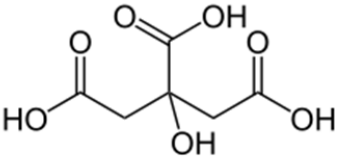 Citric acid	7	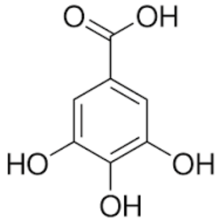 Gallic acid
3	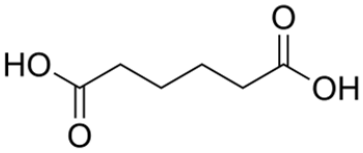 Adipic acid	8	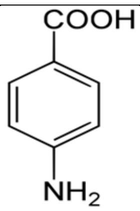 p-amino benzoic acid
4	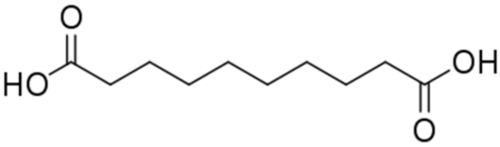 Sebacic acid	9	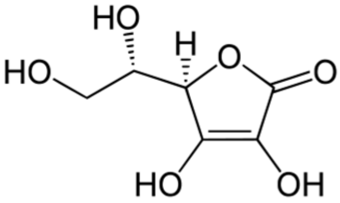 Ascorbic acid
5	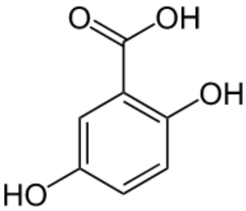 Gentisic acid	10	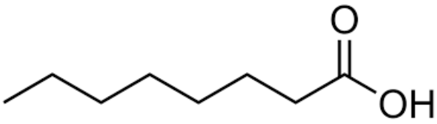 Caprylic acid

S. No	Name and structure of compound	S. No	Name and structure of compound
11	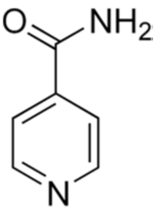<br>Isonicotinamide	*16*	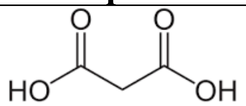<br>*Malon*ic acid
12	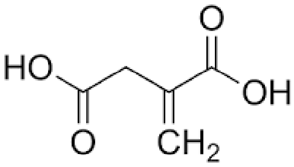<br>Itaconic acid	17	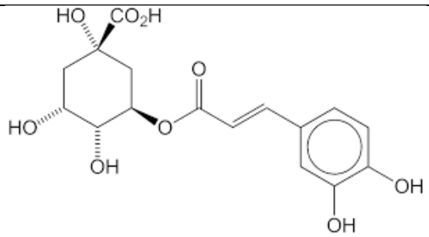<br>Chlorogenic acid
13	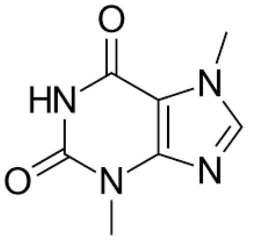<br>Theobromine	18	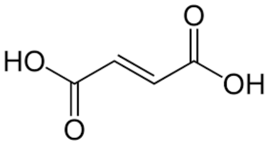<br>Fumaric acid
14	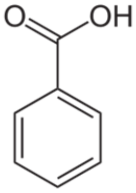<br>Benzoic acid	19	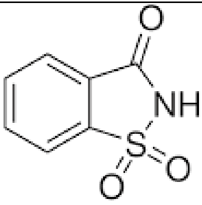<br>Saccharin
15	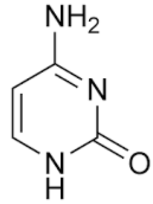<br>Cytosine	20	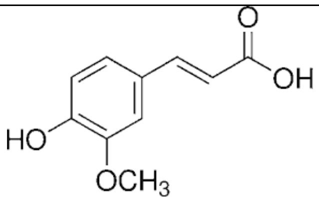<br>Ferulic acid

S. No	Name and structure of compound	S. No	Name and structure of compound
21	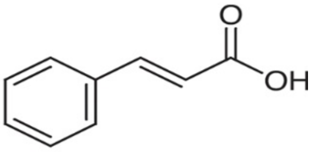 *Cinammic* acid	*26*	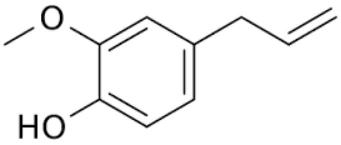 Eugenol
22	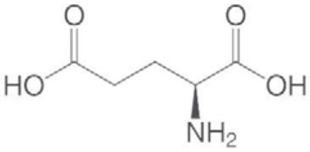 Glutamic acid	27	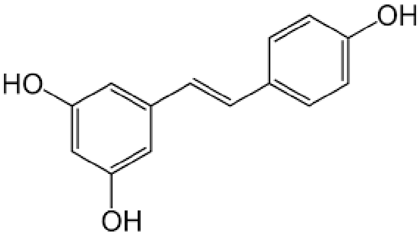 Resveratrol
23	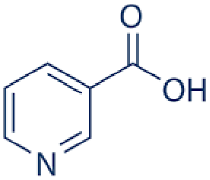 Nicotinic acid	28	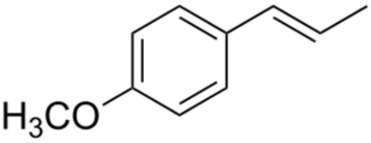 Anethole
24	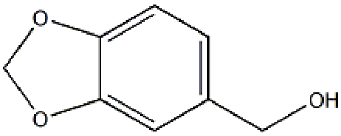 Piperonyl alcohol	29	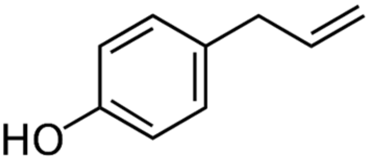 Chavicol
25	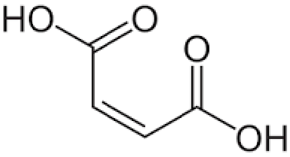 Maleic acid	30	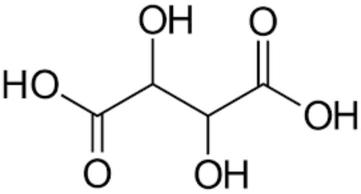 Tartaric acid
